# Supplementary material for: A cleavage rule for selection of increased-fidelity SpCas9 variants with high efficiency and no detectable off-targets
Source: Nat Commun. 2023 Sep 16;14:5746. doi: 10.1038/s41467-023-41393-5 (PMC10505190; doi:10.1038/s41467-023-41393-5)
Supplement: Supplementary file 1 — Supplementary Information [file 41467_2023_41393_MOESM1_ESM.pdf]

Summary

Supplementary Figures 2
Supplementary Figure 1 2
Supplementary Figure 2 3
Supplementary Figure 3 5
Supplementary Figure 4 7
Supplementary Figure 5 8
Supplementary Figure 6 9
Supplementary Figure 7 13
Supplementary Figure 8 14
Supplementary Figure 9 16
Supplementary Figure 10. 17
Supplementary Figure 11 21
Supplementary Tables 22
Supplementary Table 1 22
Supplementary Table 2 22
Supplementary Notes 23
Supplementary Note 1. sgRNA spacer cloning 23
Supplementary Note 2. SpCas9 variants, human expression plasmids 23
pX330-Flag-WT SpCas9 (without sgRNA; with silent mutations) (Addgene #126753)[5] 23
HypaR-SpCas9 (without sgRNA; with silent mutations) (Addgene #126757) 27
pX330-xCas9 (without sgRNA) (Addgene #) 29
Supplementary Note 3. SpCas9 variants, bacterial expression plasmids 31
Supplementary Note 4. Cloning of new B-SpCas9 variants 32
Supplementary Note 5. Cloning of in-between increased fidelity SpCas9 variants 32
Supplementary Note 6. Cloning of xCas9, SpCas9-NG and HypaR-SpCas9 variants 33
Supplementary Note 7. Other plasmid sequences 34
Prnp.HA-EGFP-DHFR[DD] 34
Sprn.HA-CMV-EGFP 37
'Self-cleaving' EGFP-expression plasmid[10] 40
Supplementary References 44

Supplementary Figures

Supplementary Figure 1

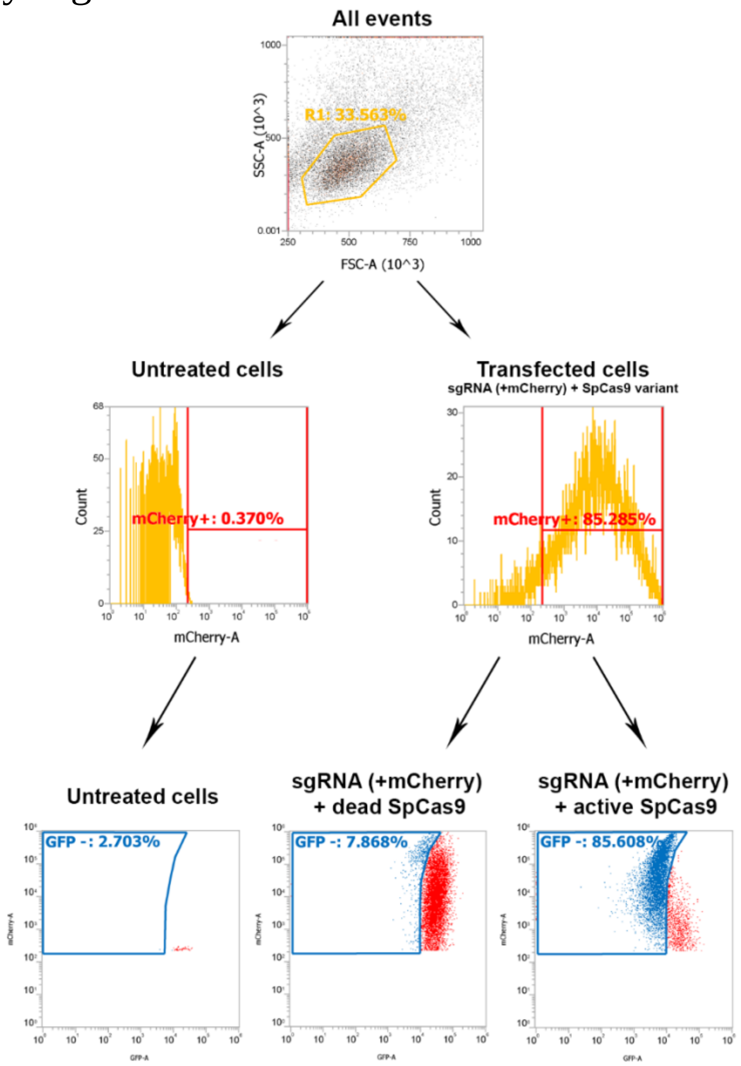

Supplementary Figure 1. Flow cytometry gating example is shown for experiments with N2a.dd-EGFP, N2a.EGFP, HEK-293.EGFP, HEK293 and GM08207 cell lines.
Single cells were gated by FSC and SSC parameters (upper panel - R1). Middle panels show mCherry FACS histograms (for R1 gated cells). Middle left panel shows an example for untreated (non-transfected) cells, middle right panel for transfected cells. In the case of HEK293 and GM08207 cells only the percentage of mCherry positive cells was determined by flow cytometry. In the case of N2a.dd-EGFP, N2a.EGFP and HEK-293.EGFP cells GFP fluorescence was measured in the mCherry positive population (lower panels). The lower panels show the gated GFP negative cells (GFP loss in EGFP disruption) in the case of untreated cells (lower left panel), cells transfected with sgRNA and mCherry coding plasmid and dead SpCas9 coding plasmid (lower middle panel) and cells transfected with sgRNA and mCherry coding plasmid and active SpCas9 variant coding plasmid (lower right panel).

Supplementary Figure 2

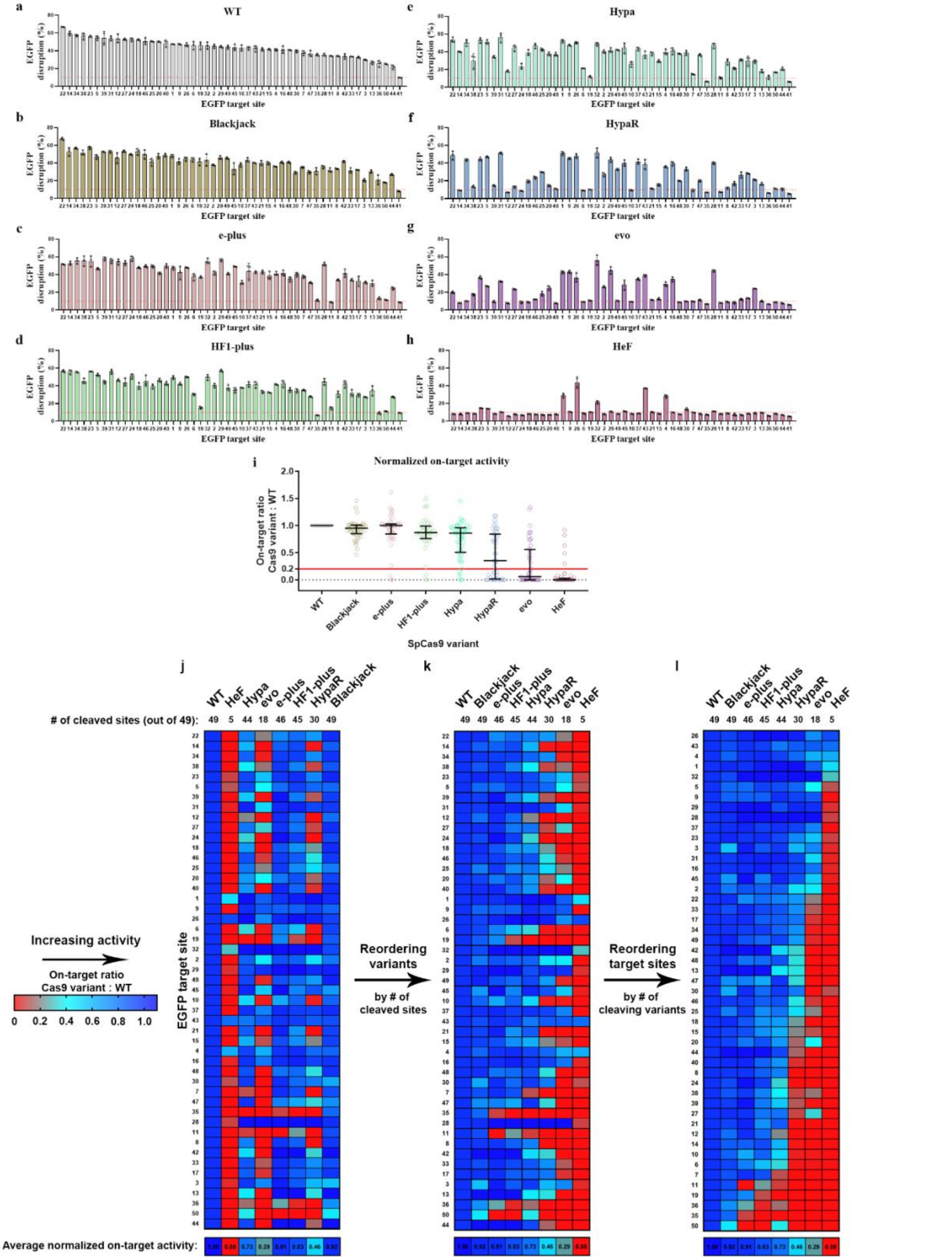

Supplementary Figure 2. The cleavage rule becomes apparent by rearranging the normalized disruption data

a-h, On-target EGFP disruption data of (**a**) WT SpCas9 and (**b-h**) seven IFN variants, as indicated in the panels, on 50 EGFP target sites. Means and SD are shown; n=3 biologically independent samples (overlaid as white circles); level of background EGFP loss is indicated by the red dashed line (average of the percentage of dead SpCas9 controls from all target sites). The targets are arranged in a descending

order based on WT disruption values in all panels. EGFP site 41 was not even cleaved by the WT SpCas9, and therefore it is not included in any downstream figures. This accounts for the difference in the number of targets (50 vs. 49) between panels (**a-h**) and other figures. **i,** On-target disruption activities normalized to the WT disruption values of different SpCas9 variants presented on a scatter dot plot. The sample points correspond to data presented in panels (**a-h**). Continuous red line indicates 0.20 normalized disruption activity, under which we consider the IFNs not to be active on a given target. The median and interquartile range are shown; data points are plotted as open circles representing the mean of biologically independent triplicates, n=49. Statistical significance was assessed by using RM one-way ANOVA and shown in Supplementary Data file 9. **j-l,** Heatmaps show the normalized EGFP disruption activity of SpCas9 nucleases with perfectly matching sgRNAs. **j**, The targets are arranged in a descending order based on WT disruption values, like in panels (**a-h**). **k,** IFNs were reordered according to how many targets they could cleave, and **l**, targets were reordered based on how many IFNs could cleave them (same pattern as in Supplementary Figure 3a). Reordering highlights the rankings amongst targets and IFNs and reveal the cleavage rule; if a target can be cleaved by a nuclease variant, then it will be cleaved by all variants with a higher activity/lower fidelity rank. **a-l**, Target sequences, raw, processed and heatmap disruption data and statistical details are reported in Supplementary Data files 1-3 and 9.

Supplementary Figure 3

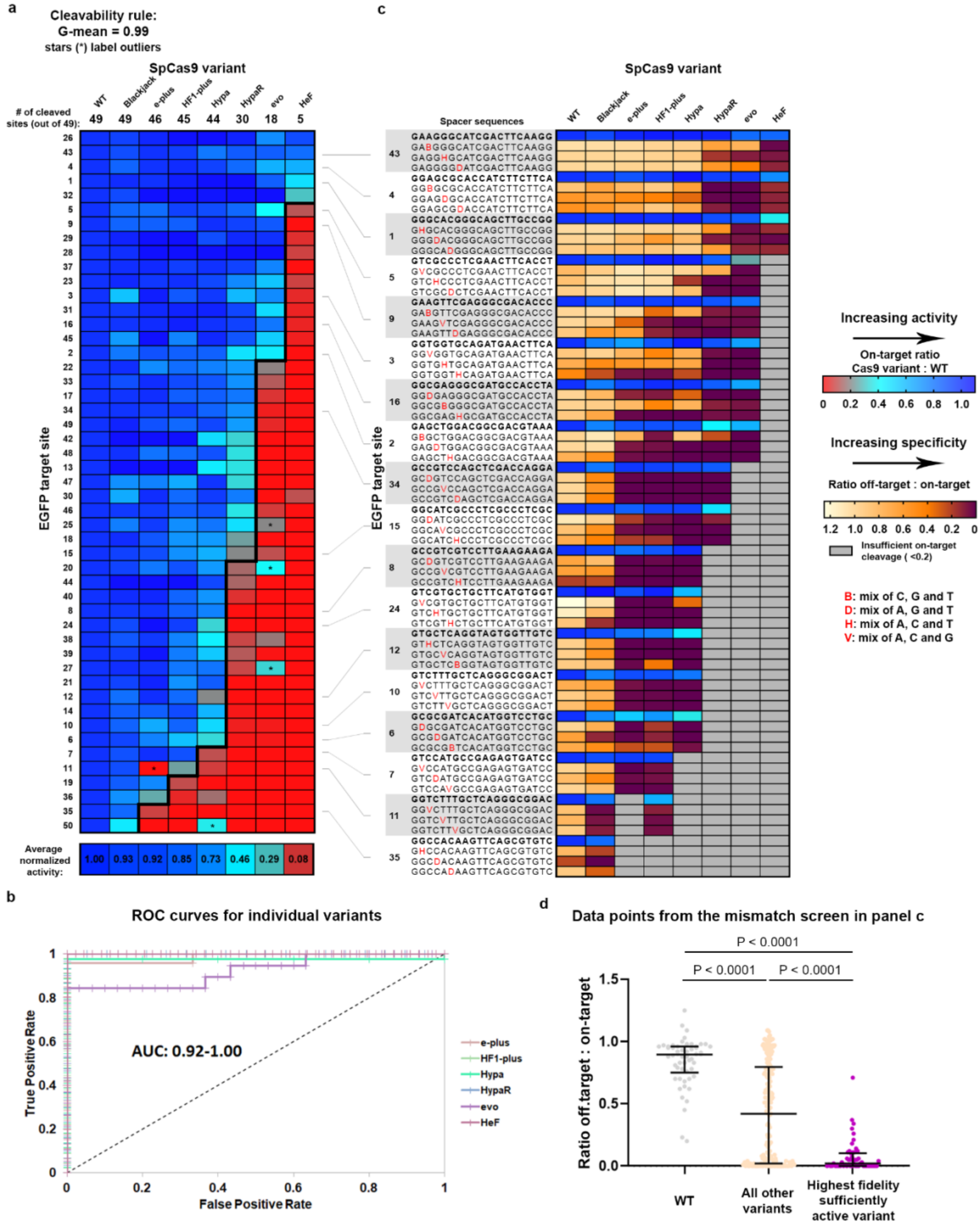

Supplementary Figure 3. Cleavage rule enables the selection of the most optimal increased-fidelity nuclease

Heatmaps show normalized EGFP disruption activities of SpCas9 nucleases with (**a**) perfectly matching and (**c**) partially mismatching 20G-sgRNAs. **a,** The bold line indicates the dividing line defined by the cleavage rule between classes of cleaved and not-cleaved values. The G-mean value indicates how well the data points above and below the bold line correspond to cleaved and not-cleaved (<0.20 activity normalized to WT) experimental values. Outliers have been confirmed in repeated experiments. **b,** The ROC curves demonstrate that the order of the target sequences, determined by the cleavage rule,

competently separates the classes of cleaved and not-cleaved normalized disruption values of each individual variant from panel (**a**). This indicates that IFNs universally perceive the target contributions that primarily determine whether they cleave a particular target or not. **c**, Mismatch screen of the nuclease variants either with perfectly matching 20G-sgRNAs (blue to red) or with one-base mismatching sgRNAs (a mixture of three different sgRNAs used for each examined mismatch position[1]; yellow to burgundy) as indicated in the figure. Grey boxes: not determined because on-target activity was too low. Targets from higher ranks (cleavable by many IFNs) require higher fidelity nucleases, while targets from lower ranks (cleavable by few IFNs) require lower fidelity nucleases for editing them with both high efficiency and high specificity. **d,** Matching IFNs and targets further increases the specificity of editing. The median and interquartile range of data points that are selected from panel (**c**) is presented as indicated; n=54, 189, 54, respectively. Dots are shown for each variant with each mismatching spacer position, where the normalized on-target activity exceeded 70%. Statistical significance was assessed by RM one-way ANOVA, statistical details and p-values are available in Methods and in Supplementary Data file 9. **a-d,** Target sequences, raw and processed disruption data and statistical details are reported in Supplementary Data files 1-4 and 9.

Supplementary Figure 4

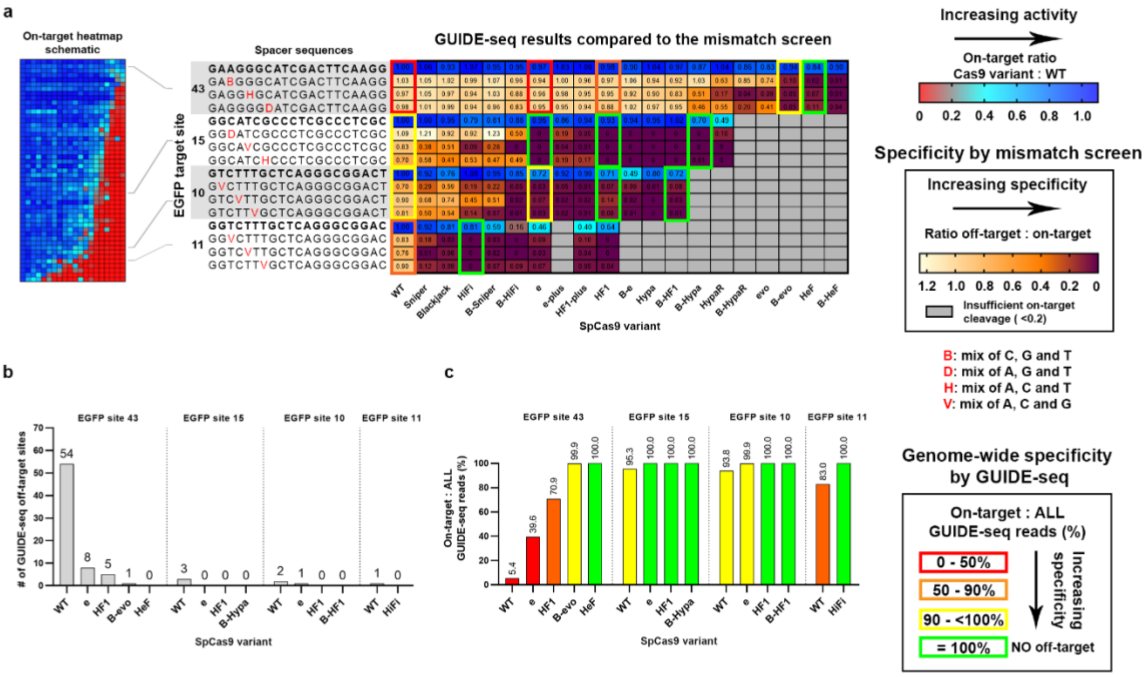

Supplementary Figure 4. The mismatch tolerance of IFNs seen in GFP disruption well approximates their genome-wide off-target effects.

a-c, Four targets and various IFNs were selected from the on-target and mismatch screen of Figure 2a and 2d and tested for genome-wide off-target events by GUIDE-seq. **a,** Genome-wide off-target data fit appropriately into the results of the mismatch screen for the targets tested. Heatmap presented here is a segment from Figure 2d. The color of the rectangular frame indicates the overall on-target specificity of an IFN on the given target as indicated in panel (**c**) (see calculations in Supplementary Data file 6). Schematical heatmap of Figure 2a on the left shows the position of the selected EGFP target sites within the ranking. **b,** Bar chart of the total number of off-target sites detected by GUIDE-seq for data shown in panels (**a**). **c,** Bar chart of the overall on-target cleavage specificity expressed by the percentages of the on-target reads captured by GUIDE-seq. **a-c,** Data is related to Supplementary Figure 5. Target sequences, raw, processed and heatmap disruption data and GUIDE-seq data are reported in Supplementary Data files 1-3 and 6.

Supplementary Figure 5

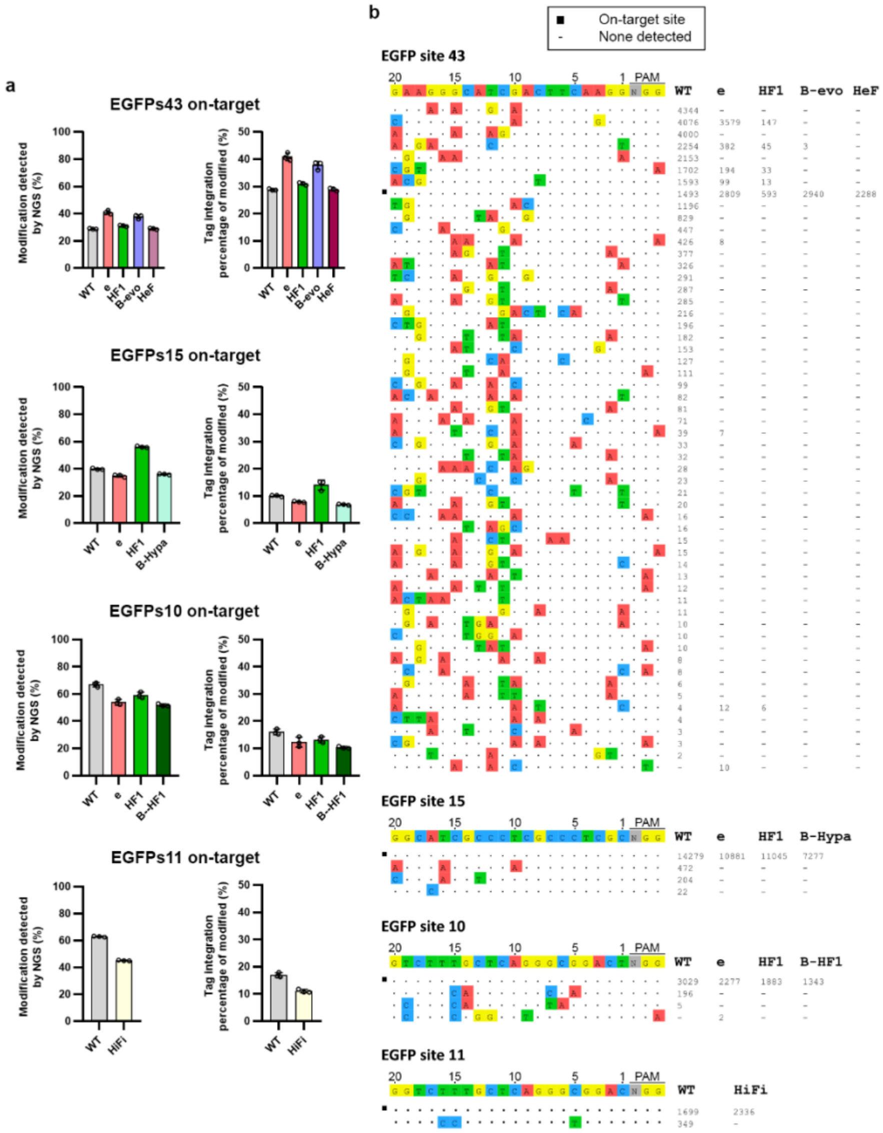

Supplementary Figure 5. Details of the GUIDE-seq experiments presented in Supplementary Figure 4

Modifications at on-target and off-target cleavage sites of SpCas9 variants with sgRNAs targeting EGFP sites identified in GUIDE-seq experiments. **a**, The percentage of on-target genome modification (indel + tag integration) and the tag integration frequency of the modified cells analyzed by NGS are presented in the bar charts. Means and SD are shown; n =3 (overlaid as white circles). **b,** Read counts that give an approximate measure of cleavage frequency at a given sequence are shown; mismatched positions within the spacer or PAM are highlighted in different colors. (-) indicates zero reads, which means that off-target cleavage was not detected; black squares indicate the on-target sites. **a-b**, Data related to Supplementary Figure 4. Target sequences, NGS and GUIDE-seq data are reported in Supplementary Data files 1, 5 and 6.

Supplementary Figure 6

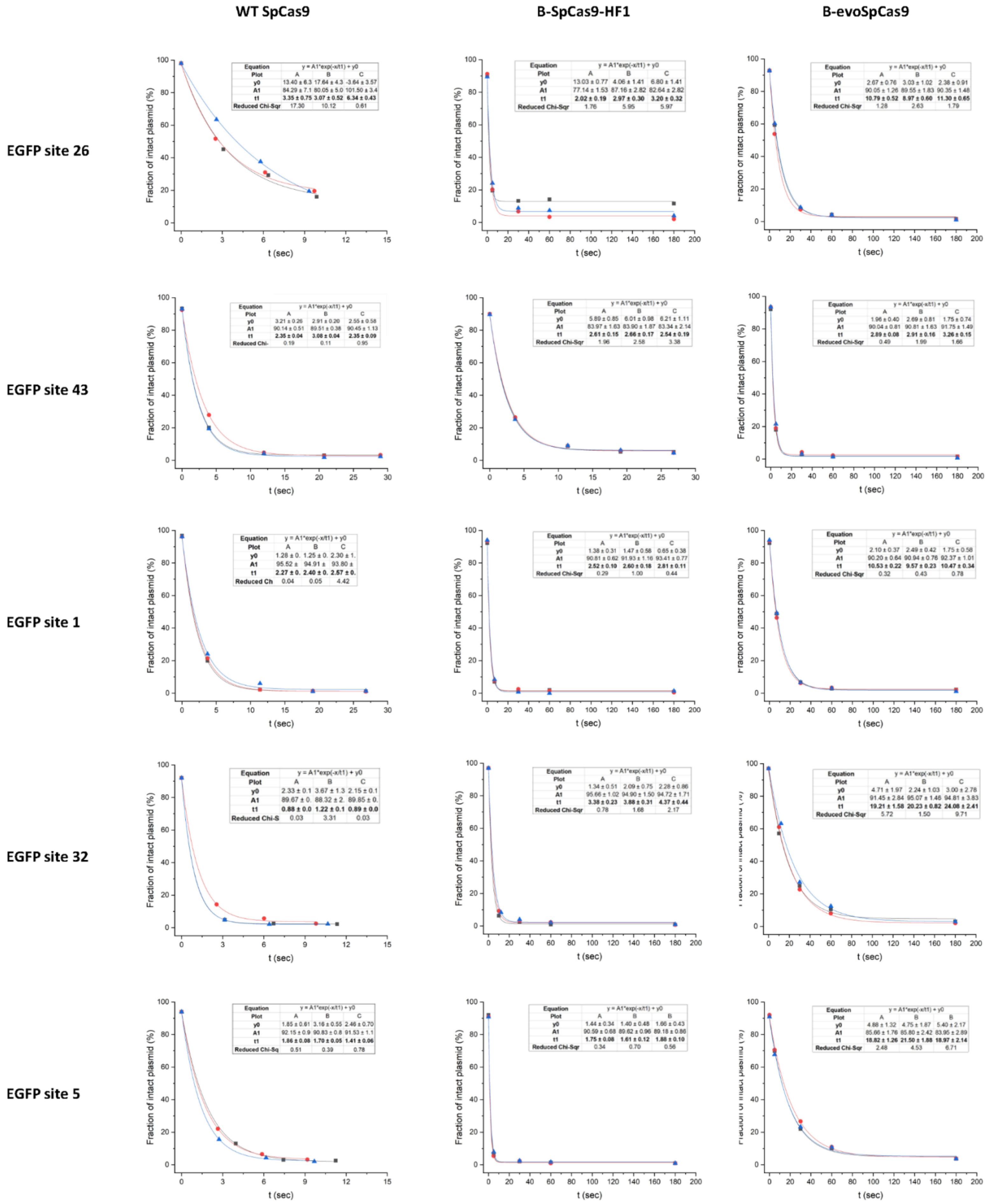

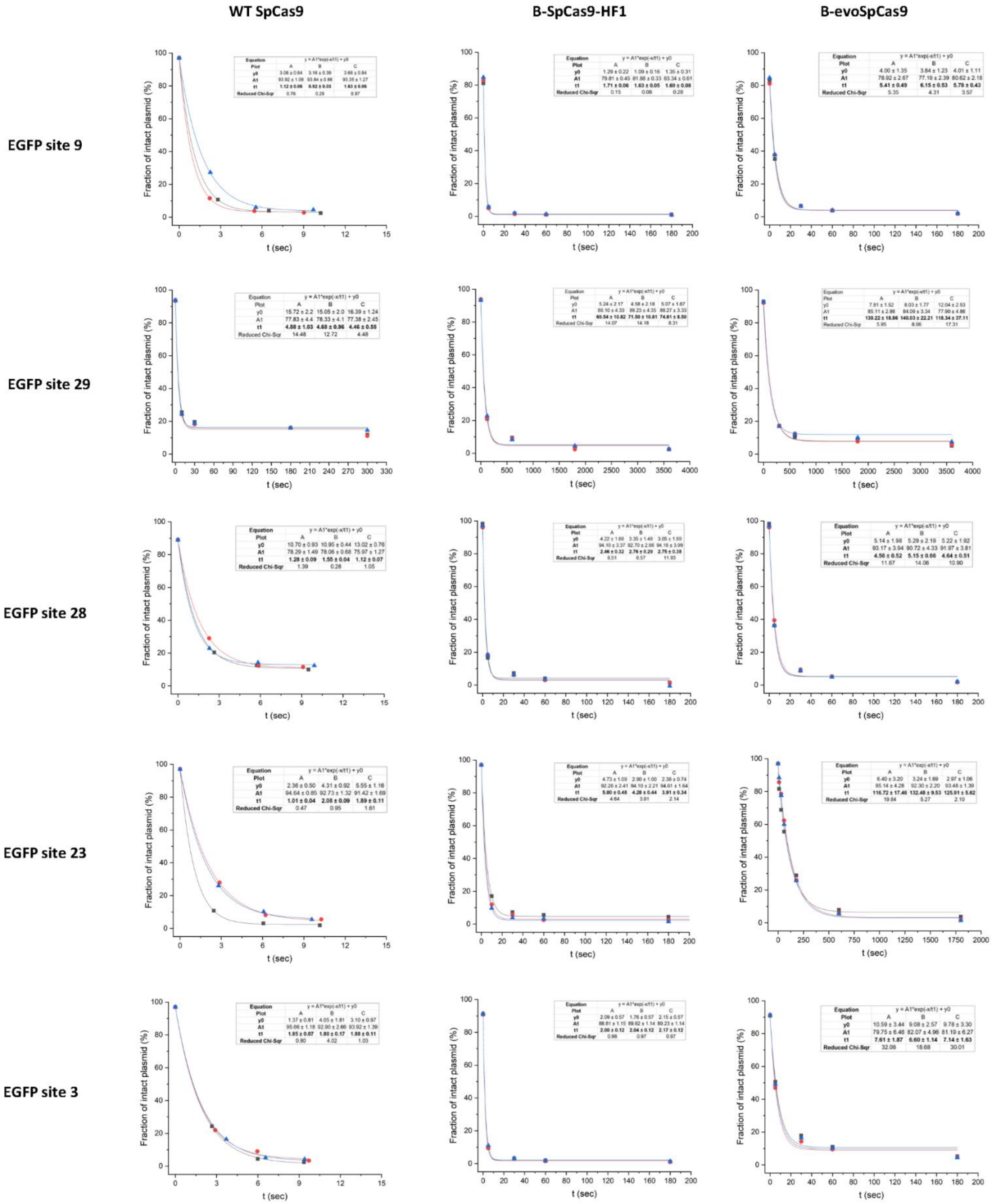
WT SpCas9
B-SpCas9-HF1
B-evoSpCas9
EGFP site 9
EGFP site 29
EGFP site 28
EGFP site 23
EGFP site 3
Fraction of intact plasmid (%)
t (sec)
y = A1*exp(-x/t1) + y0

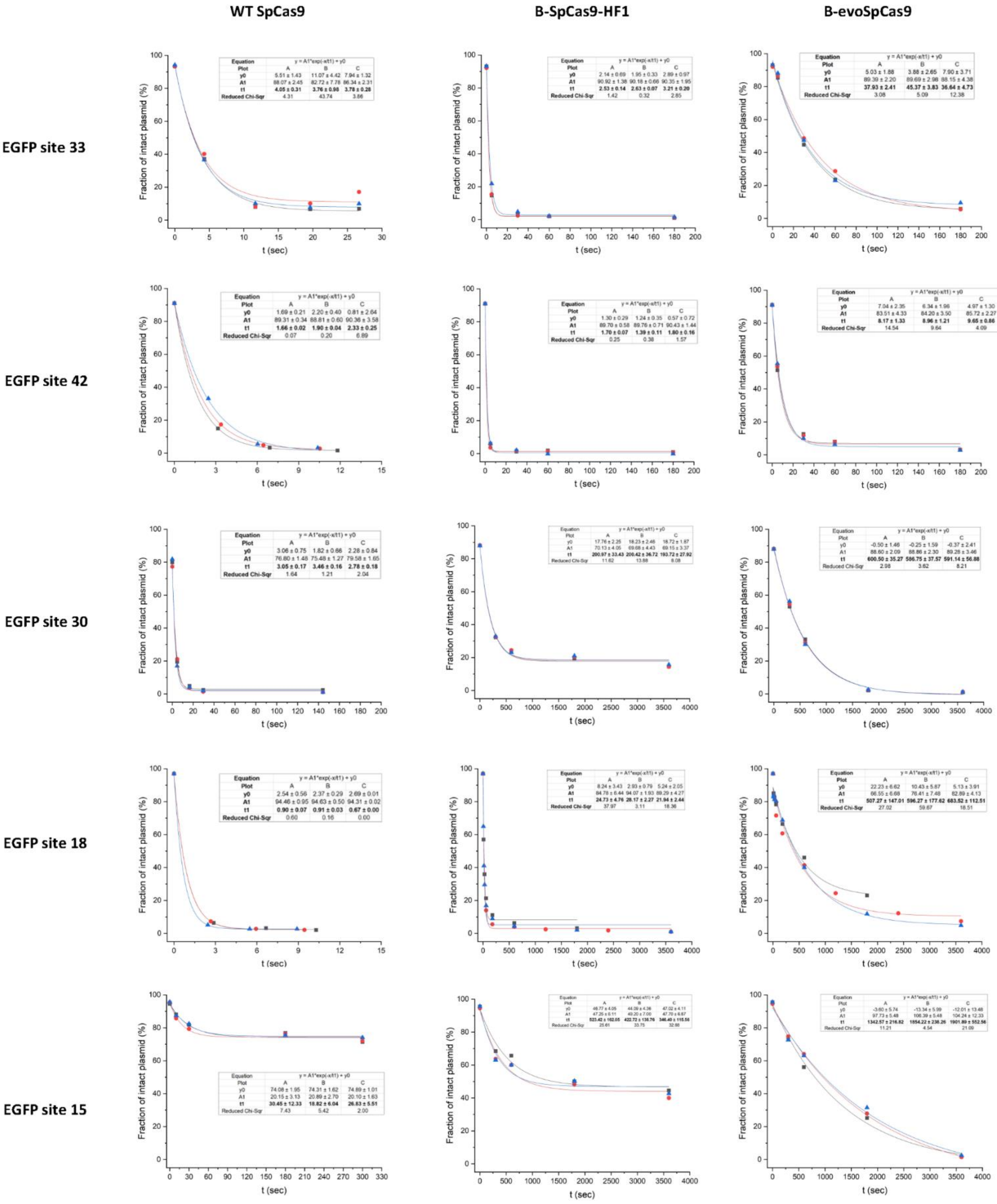
WT SpCas9
B-SpCas9-HF1
B-evoSpCas9
EGFP site 33
EGFP site 42
EGFP site 30
EGFP site 18
EGFP site 15
Fraction of intact plasmid (%)
t (sec)
Equation
y = A1*exp(-x/t1) + y0
Plot
y0
A1
t1
Reduced Chi-Sqr

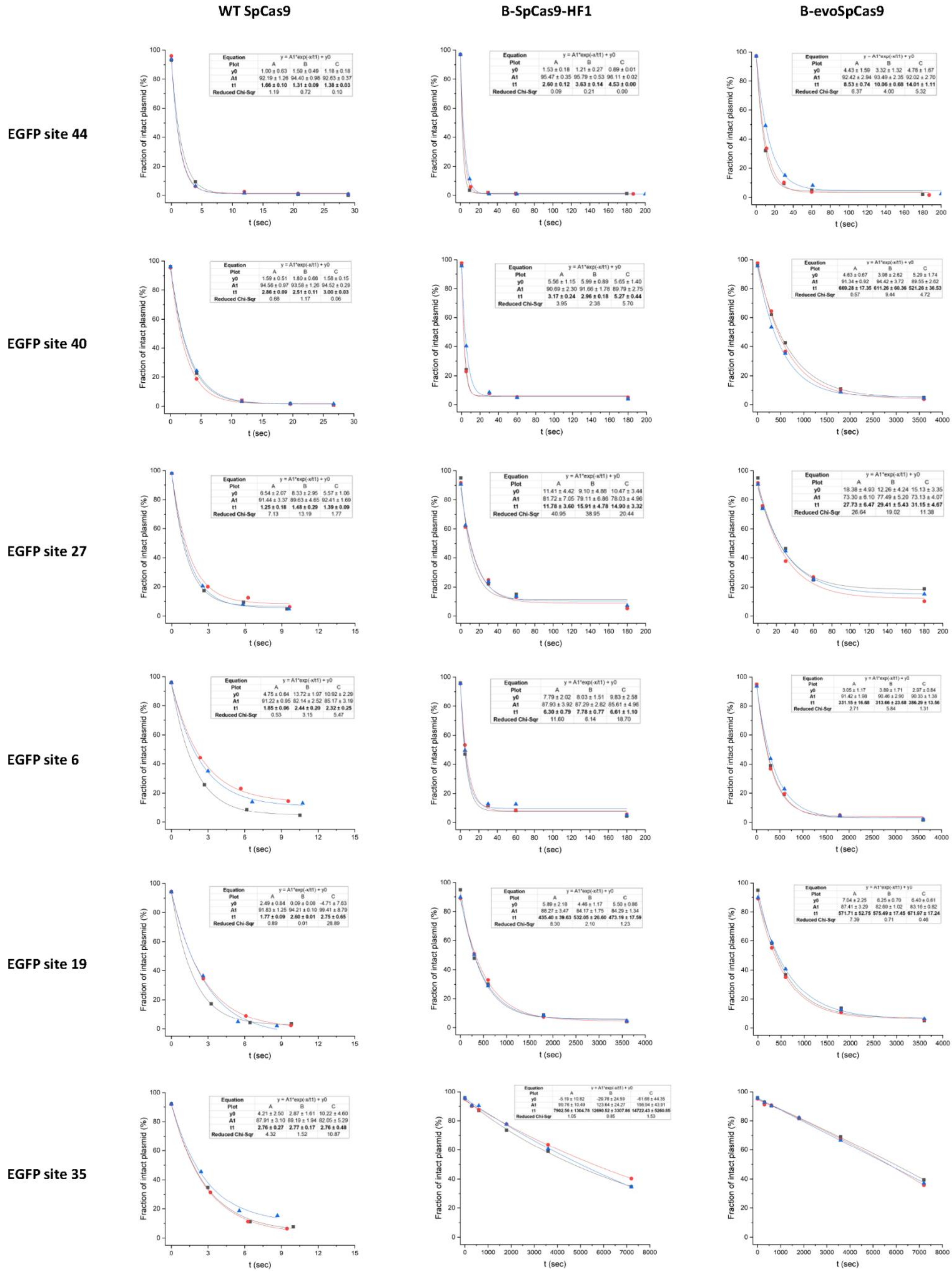

Supplementary Figure 6. ***In vitro*** **fitted curves on 21 EGFP target sites cleaved with WT, B-HF1 or B-evoSpCas9 variants**
The experiments were conducted as follows: The RNP complex was added to circular plasmid DNA (bearing the EGFP target site complementary to the spacer of the sgRNA) and the fraction of intact plasmid was measured at different time points. 0 sec was

determined by running a plasmid-only control. Plots show values derived from the band intensities measured on agarose gel. Exponential curves were fitted to the fraction of intact plasmid measured during the time frame of the experiments, in the case of each replicate separately. The values and average k values were derived from these fitted curves. Data related to Figure 4. Summary of target and primer sequences and *in vitro* data are reported in Supplementary Data files 1 and 8.

Supplementary Figure 7

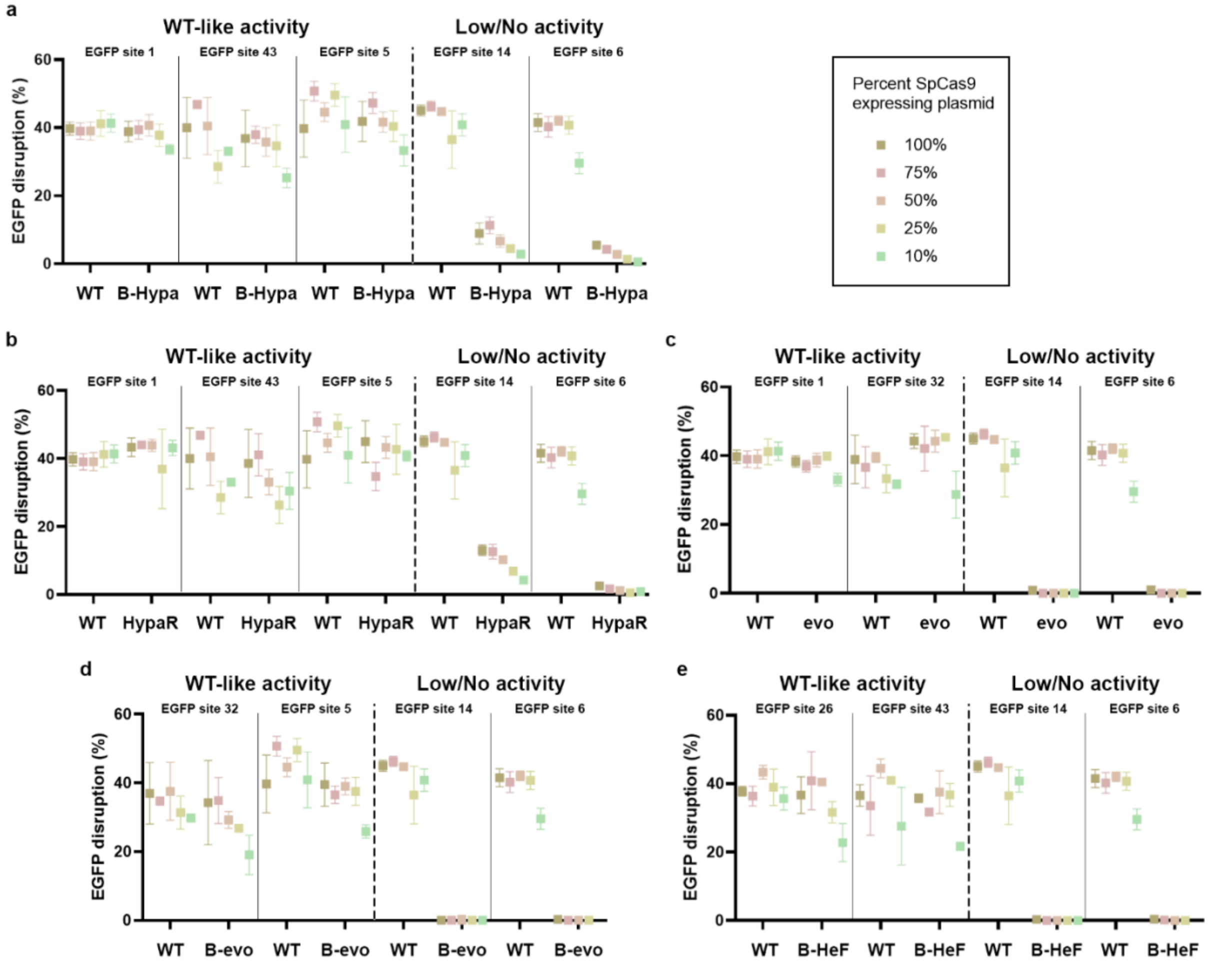

Supplementary Figure 7. IFN-coding plasmid titration

Titration of plasmid amounts expressing wild-type SpCas9 and different IFN variants on selected targets on which the IFN has either WT-like activity or no/low activity. Means are shown for each data point, error bars represent the standard deviation (SD) for triplicates. b, WT SpCas9 values are replotted from (a); c, WT SpCas9 values are replotted from (a) except EGFP site 32 data; d, WT SpCas9 values are replotted from (a) except EGFP site 32 data; e, WT SpCas9 values are replotted from (a) except EGFP site 26 and 43 data. Target sequences, raw and processed disruption data are reported in Supplementary Data files 1-3.

Supplementary Figure 8

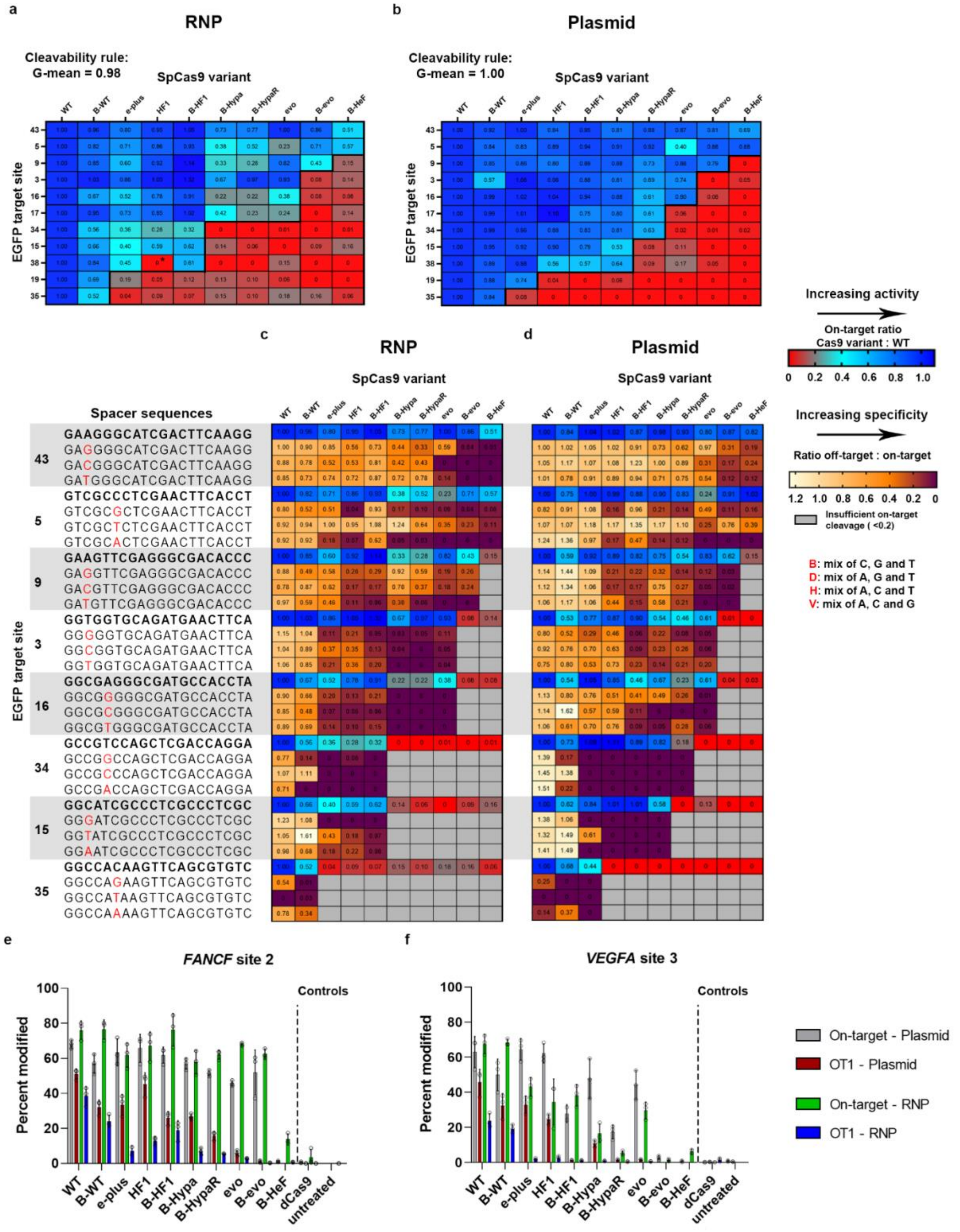

Supplementary Figure 8. The cleavage rule is also apparent and an identical order of targets and IFNs is observed with the RNP as with the plasmid form when tested with 9 IFNs.

a-b, Heatmaps show the normalized EGFP disruption activities of SpCas9 nucleases with perfectly matching 20G-sgRNAs in case of RNP (**a**) or plasmid (**b**) form. The bold line indicates the dividing line defined by the cleavage rule between the classes of cleaved and not-cleaved values. **c-d**, Heatmaps show the normalized EGFP disruption activities of SpCas9 nucleases either with perfectly matching (blue to

red) or with one-base mismatching (a mixture of three different sgRNAs used for each examined mismatch position[1]; yellow to burgundy) 20G-sgRNAs in case of RNP (**c**) or plasmid (**d**) form. Grey boxes: not determined because on-target activity was too low. **e-f**, Bar chart of the results of on-target and the top off-target site detected by NGS in case of WT and nine IFNs alongside with various negative controls. Means and SD are shown; n=3 (overlaid as white circles). Target and primer sequences, raw and processed disruption and NGS data are reported in Supplementary Data files 1-5.

Supplementary Figure 9

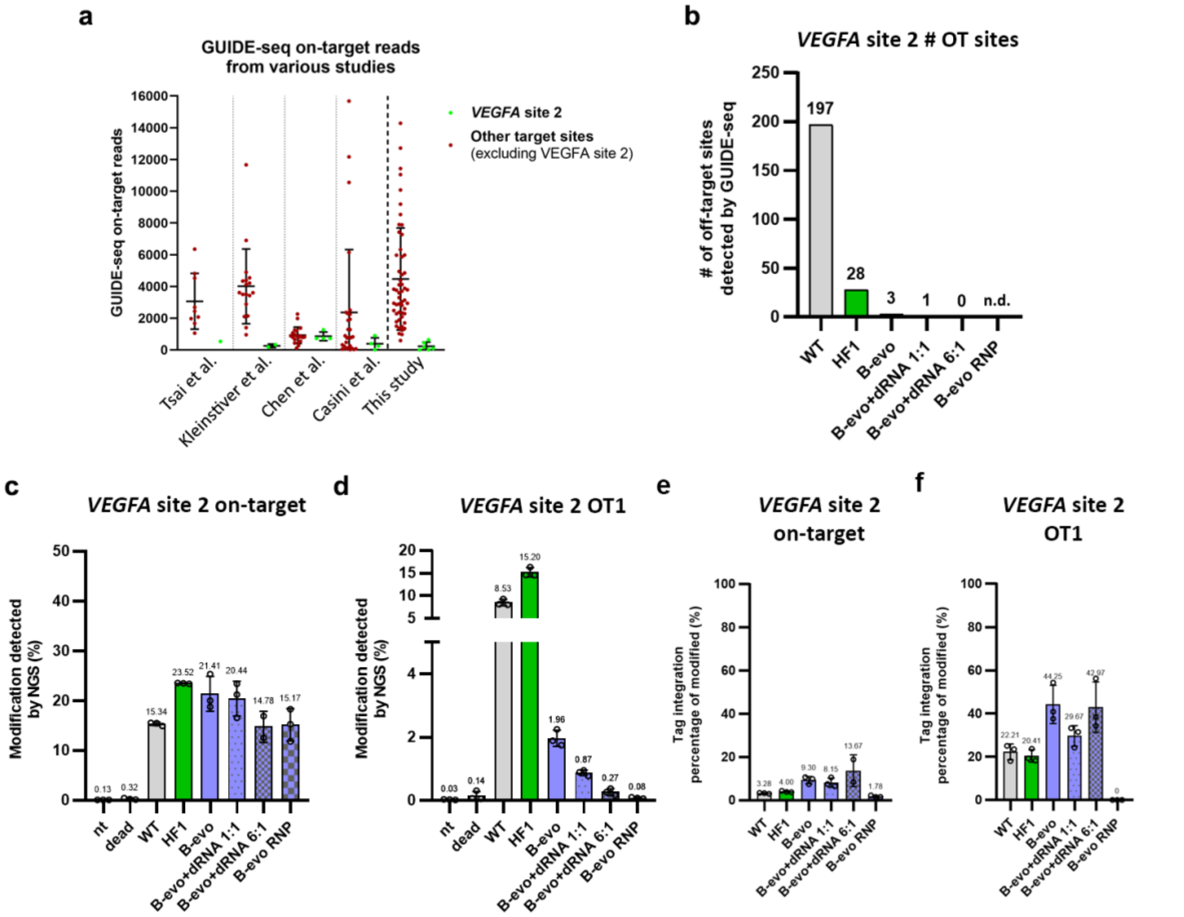

Supplementary Figure 9. NGS confirms the editing of *VEGFA* site 2 without any off-target
a, Scatter plot shows that the mean and SD of the on-target GUIDE-seq reads for all targets used in a given study (for details see Supplementary Data file 6) is the highest in this study, stressing the significance of the editing without any off-targets demonstrated here. The on-target GUIDE-seq reads for *VEGFA* site 2 target are unusually low across all studies compared to most other target sites preventing a straightforward interpretation of the off-target reads. In the scatter dot plot means and SD are shown; data points are plotted as circles representing the on-target GUIDE-seq reads in the case of every tested target site in a given paper. **b-f,** NGS confirms the editing of *VEGFA* site 2 without any off-target. **b,** Bar chart showing the total number of off-target sites detected by GUIDE-seq. Due to the few on-target reads for *VEGFA* site 2, we performed amplicon sequencing using the samples of panel (**b**) to sequence the (**c**) on-target site and the (**d**) off-target site 1 (OT1), its most persistent off-target sequence. **c, d,** The percentage of genome modification (indel + tag integration) is shown in bar charts. Means and SD are shown; n=3 (overlaid as white circles). **b-f,** While on-target modification rates are comparable with the WT SpCas9 in all conditions in panel (**c**), OT1 reads in panel (**d**) are decreasing in accordance with the total number of off-target sites detected by GUIDE-seq (**b**) and with the GUIDE-seq reads in Supplementary Figure 11**.** The combination of B-evoSpCas9 with dRNA[2] (B-evo+dRNA 6:1) diminishes off-target editing detected by GUIDE-seq although showing detectable OT1 modification by NGS as shown in panel (**b**) and in panel (**f**), and low on-target GUIDE-seq read counts (Supplementary Fig. 11). In panel (**b**), in case of B-evoSpCas9 in RNP form no GUIDE-seq read was detected (n.d.) even though it showed WT-like on-target cleavage (**c**) and tag integration (**e**). B-evoSpCas9 in RNP form showed a modification rate on OT1 site, that is smaller than that of dead SpCas9, falling under the detection limit of NGS (**d**) and showed no tag integration (**f**). **c-f,** The percentage of on-target genome modification (indel + tag integration) and the tag integration frequency of the modified cells are shown in bar charts, as indicated in the figure. Means and SD are shown; n=3 (overlaid as white circles). **a-f,** Data related to Figures 6-9 and Supplementary Figures 5and 11. Target sequences, NGS and GUIDE-seq data are reported in Supplementary Data files 1, 5 and 6.

Supplementary Figure 10.

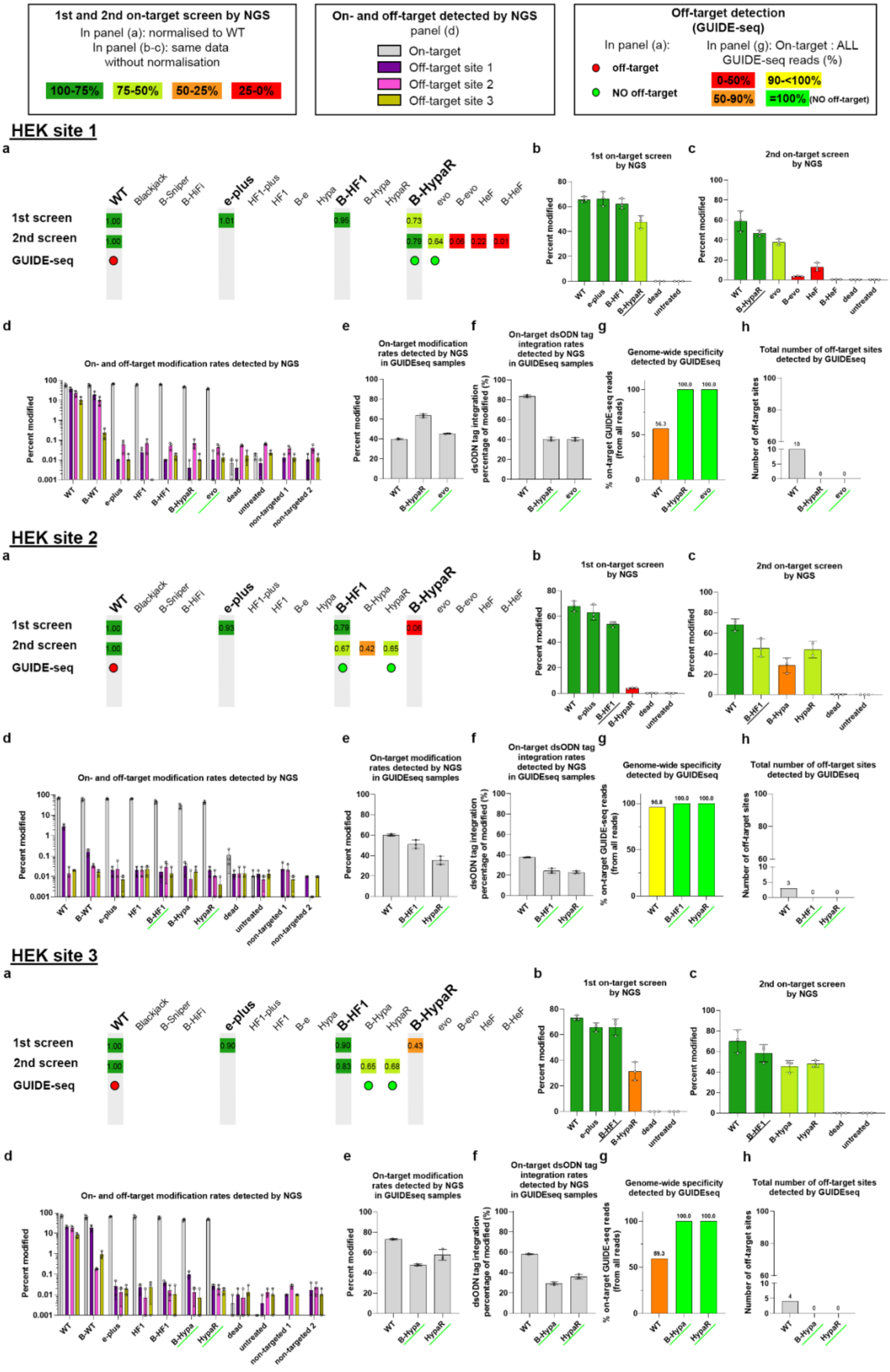

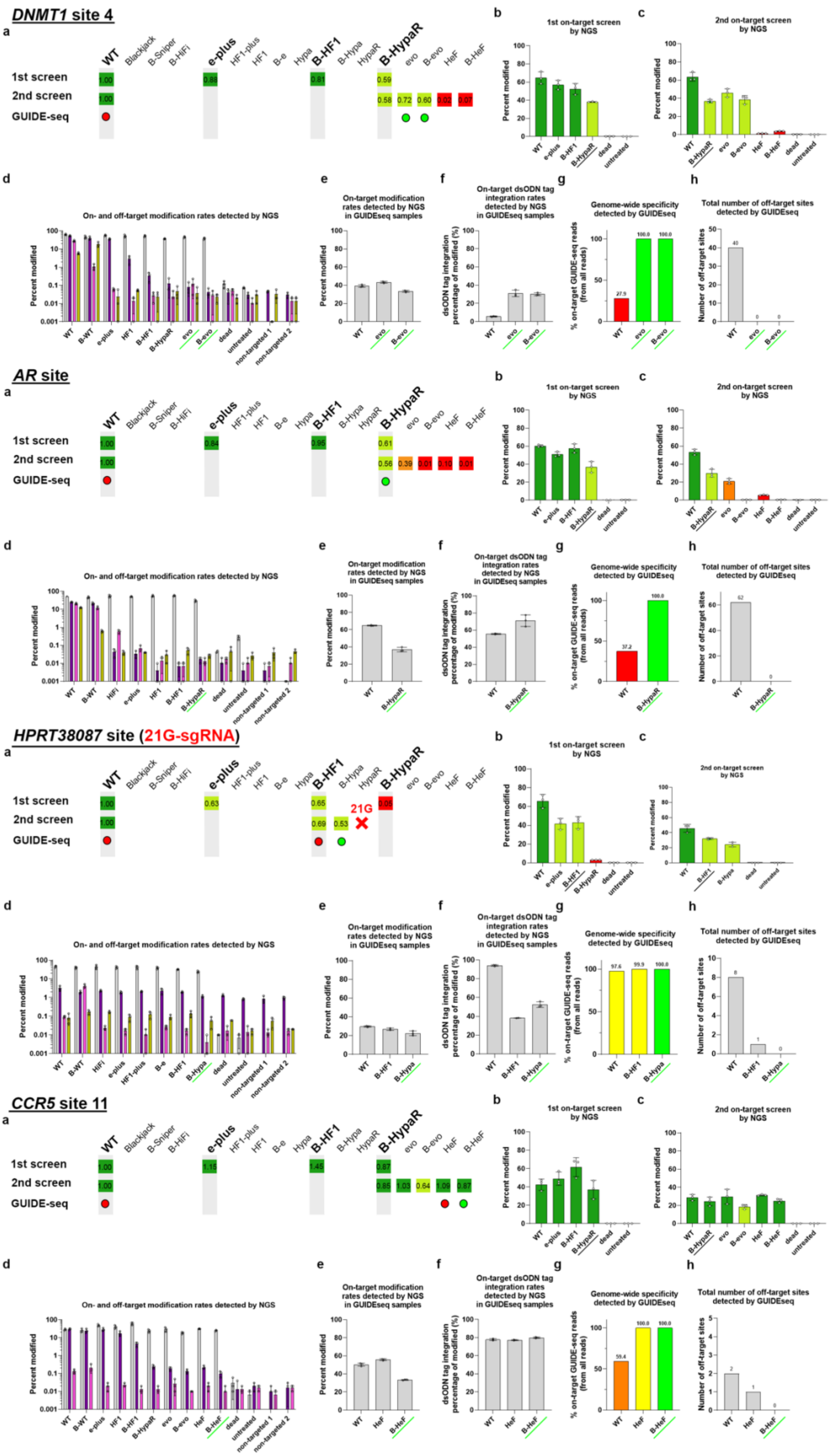
DNMT1 site 4
a
WT
Blackjack
B-Sniper
B-HiFi
e-plus
HF1-plus
HF1
B-e
Hypa
B-HF1
B-Hypa
HypaR
B-HypaR
evo
B-evo
HeF
B-HeF
1st screen
2nd screen
GUIDE-seq
b
1st on-target screen by NGS
c
2nd on-target screen by NGS
Percent modified
d
On- and off-target modification rates detected by NGS
e
On-target modification rates detected by NGS in GUIDEseq samples
f
On-target dsODN tag integration rates detected by NGS in GUIDEseq samples
dsODN tag integration percentage of modified (%)
g
Genome-wide specificity detected by GUIDEseq
% on-target GUIDE-seq reads (from all reads)
h
Total number of off-target sites detected by GUIDEseq
Number of off-target sites
AR site
HPRT38087 site (21G-sgRNA)
21G
CCR5 site 11

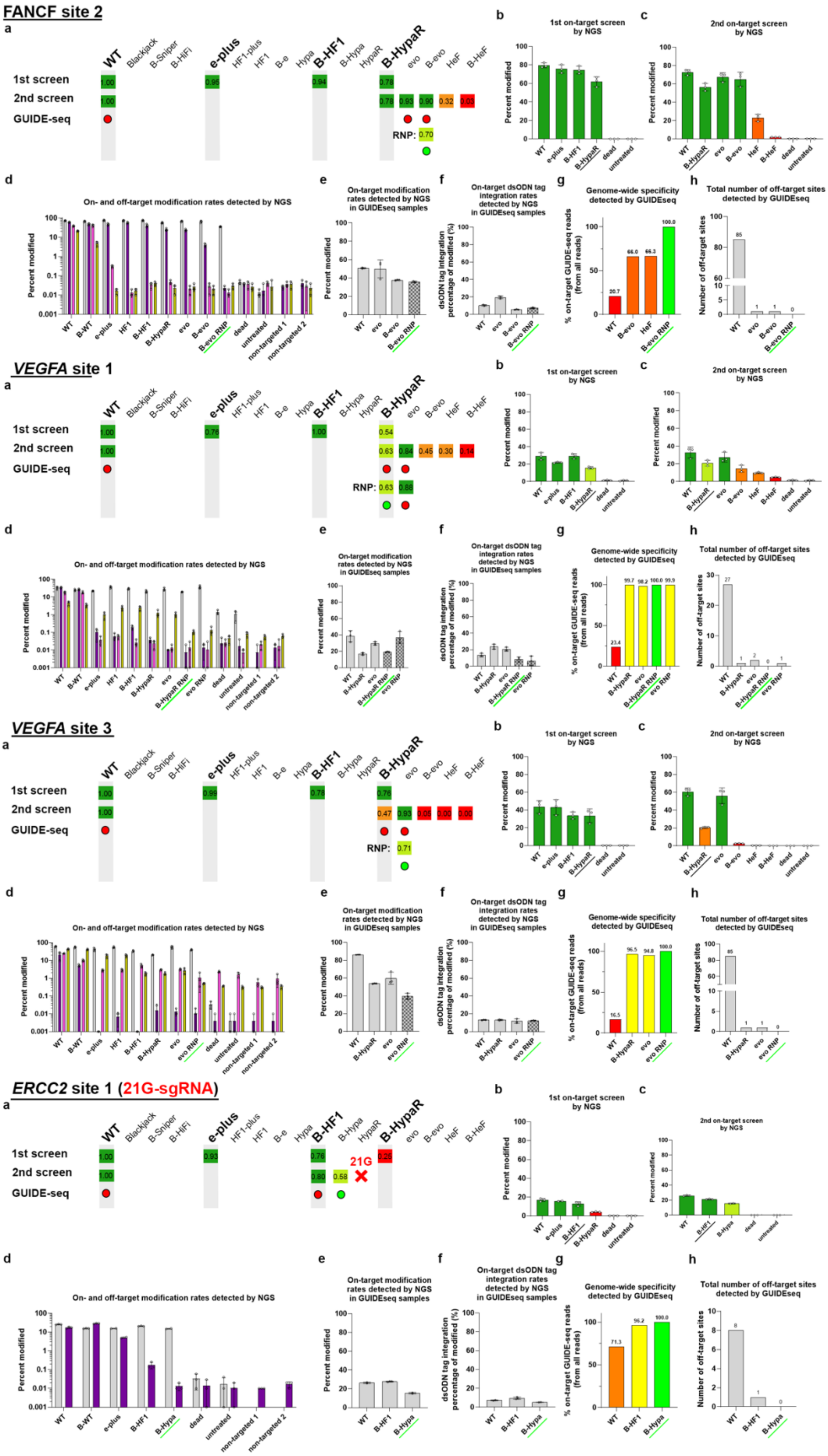
FANCF site 2
a
WT
Blackjack
B-Sniper
B-HiFi
e-plus
HF1-plus
HF1
B-e
Hypa
B-HF1
B-Hypa
HypaR
B-HypaR
evo
B-evo
HeF
B-HeF
1st screen
2nd screen
GUIDE-seq
RNP:
b
1st on-target screen by NGS
c
2nd on-target screen by NGS
Percent modified
d
On- and off-target modification rates detected by NGS
e
On-target modification rates detected by NGS in GUIDEseq samples
f
On-target dsODN tag integration rates detected by NGS in GUIDEseq samples
dsODN tag integration percentage of modified (%)
g
Genome-wide specificity detected by GUIDEseq
% on-target GUIDE-seq reads (from all reads)
h
Total number of off-target sites detected by GUIDEseq
Number of off-target sites
VEGFA site 1
VEGFA site 3
ERCC2 site 1 (21G-sgRNA)
21G

Supplementary Figure 10. Overview of on-and off-target modification ratios detected by amplicon sequencing and GUIDE-seq experiments for genomic targets tested in the study

For each target a panel set is shown: **a**, The numbers in the colored squares indicate the percentage value of the on-target genome modification normalized to WT (measured by NGS). **b, c,** Bar chart of the results of the 1st, rough on-target screen (**b**) and the 2nd, fine-tuning on-target screen (**c**). Coloring and values correspond to panel (a). **d,** Bar chart of the results of on-target and of the top three (if available) off-target sites measured by NGS in case of WT, in-between and target-matched IFN variants alongside with various negative controls. Zero values are displayed as 0.001 for logarithmic presentation. **e-f,** The percentage of on-target genome modification (indel + tag integration) and only the tag integration frequency of the modified cells in GUIDE-seq experiments are shown in bar charts, as indicated in the figure. **g,** Bar chart of the overall on-target cleavage specificity expressed by the percentages of the on-target reads among all reads captured by GUIDE-seq. **h,** Bar chart of the total number of off-target sites detected by GUIDE-seq. **b-f,** Means and SD are shown; n=3 (overlaid as white circles). **a-h,** Data related to Figures 6-9 and Supplementary Figure 11. Summary of NGS, GUIDE-seq and statistical data are reported in Supplementary Data files 5, 6 and 9.

Supplementary Figure 11

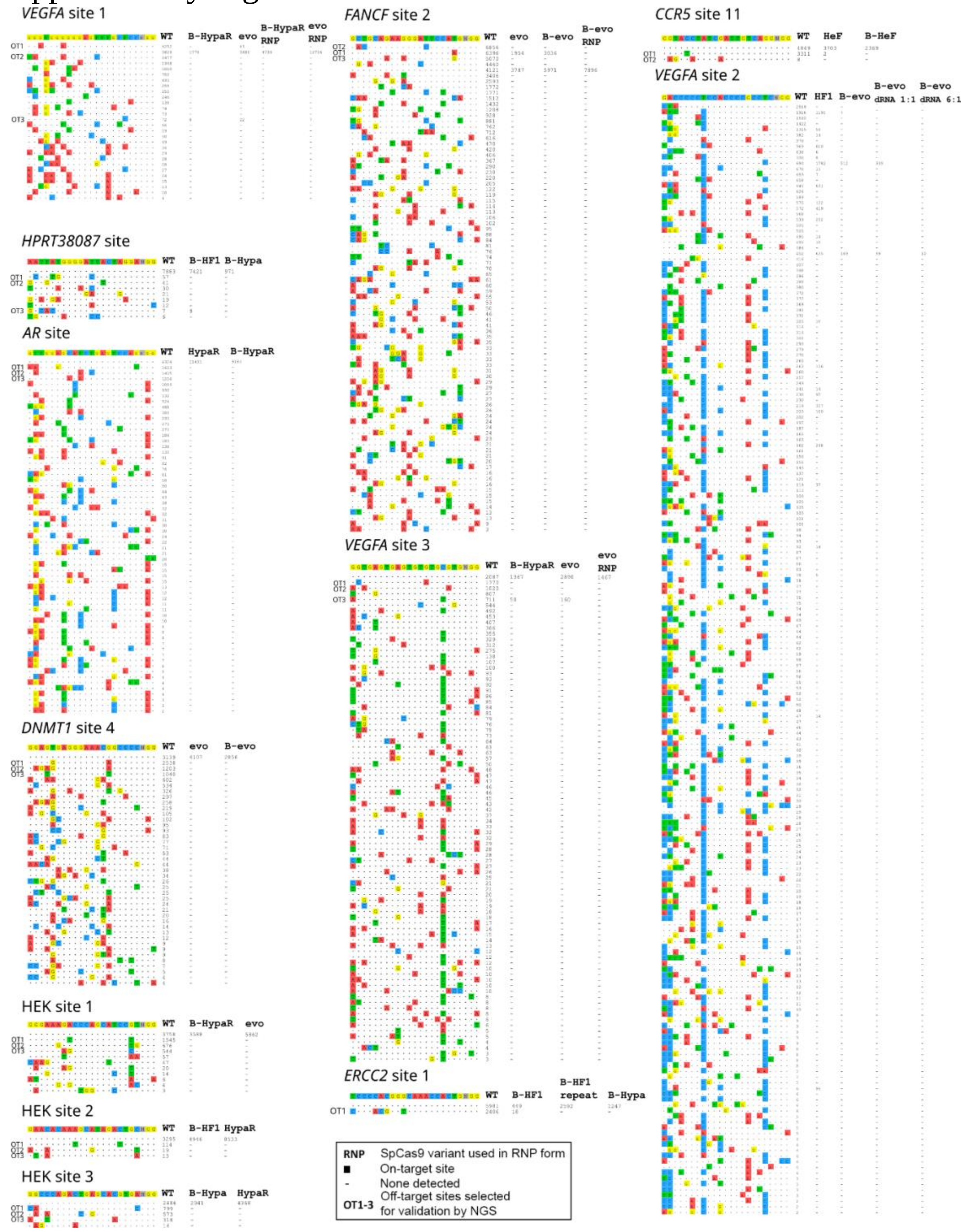

Supplementary Figure 11. Genome-wide off-target detection of increased fidelity SpCas9 variants using GUIDE-seq

Off-target cleavage sites of SpCas9 variants identified by GUIDE-seq. Read counts that give an approximate measure of cleavage frequency at a given sequence are shown, mismatched positions within the spacer or the PAM are highlighted in different colors. (-) indicates zero reads, which means that off-target cleavage was not detected, black squares indicate on-target sites, data with no GUIDE-seq read are not shown (*VEGFA* site 2 B-evoSpCas9 in RNP form). OT1-3 sites indicate selection for NGS-validation. Data related to Figures 6, 7, 9 and Supplementary Figures 9, 10. Summary of GUIDE-seq data is reported in Supplementary Data file 6.

Supplementary Tables

Supplementary Table 1

Binary classifiers of two predictions (DeepSpCas9[3] and the one developed in this study, DeepRank) of on-target cleavage activity of 5 IFNs on 49 targets from Figure 2a. For calculation details see Supplementary Data file 7: EGFP prediction and Methods: EGFP prediction.

SpCas9 variants	G-mean		Specificity		Sensitivity	
	DeepSpCas9	DeepRank	DeepSpCas9	DeepRank	DeepSpCas9	DeepRank
Sniper SpCas9	0	0	0	0	1	1
eSpCas9	0	0	0	0	1	1
SpCas9-HF1	0,63	0,45	0,4	0,2	0,98	1
HypaSpCas9	0,71	0,41	0,5	0,17	1	1
evoSpCas9	0,64	0,71	0,44	0,5	0,94	1

Supplementary Table 2

Summary of potential off-target sites identified by Cas-OFFinder[4] in the reference human genome for the sgRNAs examined in this study by GUIDE-seq. Data related to Figure 8.

	mismatches to on-target site						
site	1	2	3	4	5	6	total
EGFP site 43	0	0	5	88	827	5895	6815
CCR5 site 11	0	1	5	125	1391	10111	11633
VEGFA site 2	0	1	12	143	1519	10877	12552
FANCF site 2	0	2	35	456	3905	17576	21974
DNMT1 site 4	1	1	29	235	2000	13047	15313
VEGFA site 3	0	2	17	251	1732	11335	13337
HEK site 1	0	0	9	96	1065	7675	8845
VEGFA site 1	0	1	11	88	857	6401	7358
AR site	1	4	35	281	2202	14064	16587
HEK site 3	1	1	0	20	273	2807	3102
ERCC2 site 1	1	17	383	6089	13536	35901	55927
EGFP site 15	0	3	10	158	1377	9833	11381
EGFP site 10	0	0	2	78	875	6790	7745
HEK site 2	0	3	16	340	3153	13510	17022
HPRT38087 site	0	0	4	62	731	5649	6446
EGFP site 11	0	0	6	69	1021	8733	9829

Supplementary Notes

For detailed primer, oligonucleotide and SpCas9 construct information see Supplementary Data file 1. The sequences of all plasmid constructs were confirmed by Sanger sequencing.

Supplementary Note 1. sgRNA spacer cloning

sgRNA expression plasmids were constructed by ligating annealed DNA oligonucleotides harbouring the spacer sequence with 4 nt long overhangs into a *Bbs*I restriction enzyme digested pmCherry_gRNA (#80457), or pmCherry_gRNA_ver2 (Addgene #126776; this plasmid backbone lacks a truncated extra guideRNA scaffold sequence)[5] plasmids. A one-pot digestion-ligation protocol was followed[6]. The synthetic DNA oligonucleotides were hybridized, and the annealed oligonucleotides (2.5 µM) were mixed with 50 ng plasmid DNA, 3 units of *Bbs*I restriction enzyme and 1.5 units of T4 DNA ligase in Green buffer (Thermo Fisher Scientific) containing 500 µM ATP. The mixture was kept at 37 °C for one hour before transforming into chemically competent Stable Competent *E. coli* cells (NEB). Two single colonies, formed after culturing on an agar plate, were tested by restriction enzyme digestion and appropriate clones were sent for sequencing.

Supplementary Note 2. SpCas9 variants, human expression plasmids

pX330-Flag-WT SpCas9 (without sgRNA; with silent mutations) (Addgene #126753)[5]

The backbone of all coding plasmids of SpCas9 variants are identical to the sequence shown hereunder.

Whole DNA sequence of the plasmid:
Human codon optimized *S. pyogenes* Cas9 shown in purple, NLS underlined, 3xFLAG tag in *light blue*.
CTAGAGGTACCCGTTACATAACTTACGGTAAATGGCCCGCCTGGCTGACCGCCCAACGAC
CCCCGCCCATTGACGTCAATAGTAACGCCAATAGGGACTTTCCATTGACGTCAATGGGTG
GAGTATTTACGGTAAACTGCCCACTTGGCAGTACATCAAGTGTATCATATGCCAAGTACG
CCCCCTATTGACGTCAATGACGGTAAATGGCCCGCCTGGCATTGTGCCCAGTACATGACC
TTATGGGACTTTCCTACTTGGCAGTACATCTACGTATTAGTCATCGCTATTACCATGGTCG
AGGTGAGCCCCACGTTCTGCTTCACTCTCCCCATCTCCCCCCCCTCCCCACCCCCAATTTT
GTATTTATTTATTTTTTAATTATTTTGTGCAGCGATGGGGGCGGGGGGGGGGGGGGGGCG
CGCGCCAGGCGGGGCGGGGCGGGGCGAGGGGCGGGGCGGGGCGAGGCGGAGAGGTGCG

GCGGCAGCCAATCAGAGCGGCGCGCTCCGAAAGTTTCCTTTTATGGCGAGGCGGCGGCG
GCGGCGGCCCTATAAAAAGCGAAGCGCGCGGCGGGCGGGAGTCGCTGCGACGCTGCCTT
CGCCCCGTGCCCCGCTCCGCCGCCGCCTCGCGCCGCCCGCCCCGGCTCTGACTGACCGCG
TTACTCCCACAGGTGAGCGGGCGGGACGGCCCTTCTCCTCCGGGCTGTAATTAGCTGAGC
AAGAGGTAAGGGTTTAAGGGATGGTTGGTTGGTGGGGTATTAATGTTTAATTACCTGGAG
CACCTGCCTGAAATCACTTTTTTTCAGGTTGGACCGGTGCCACCATG*GACTATAAGGACCAC*
*GACGGAGACTACAAGGATCATGATATTGATTACAAAGACGATGACGATAAG*ATGGCCCCAAAG
AAGAAGCGGAAGGTCGGTATCCACGGAGTCCCAGCAGCCGACAAGAAGTACAGCATCGG
CCTGGACATCGGCACCAACTCTGTGGGCTGGGCCGTGATCACCGACGAGTACAAGGTGCC
CAGCAAGAAATTCAAGGTGCTGGGCAACACCGACCGGCACAGCATCAAGAAGAACCTGA
TCGGAGCCCTGCTGTTCGACAGCGGCGAAACAGCCGAGGCCACCCGGCTGAAGAGAACC
GCCAGAAGAAGATACACCAGACGGAAGAACCGGATCTGCTATCTGCAAGAGATCTTCAG
CAACGAGATGGCCAAGGTGGACGACAGCTTCTTCCACAGACTGGAAGAGTCCTTCCTGGT
GGAAGAGGATAAGAAGCACGAGCGGCACCCCATCTTCGGCAACATCGTGGACGAGGTGG
CCTACCACGAGAAGTACCCCACCATCTACCACCTGAGAAAGAAACTGGTGGACAGCACC
GACAAGGCCGACCTGCGGCTGATCTATCTGGCCCTGGCCCACATGATCAAGTTCCGGGGC
CACTTCCTGATCGAGGGCGACCTGAACCCCGACAACAGCGACGTGGACAAGCTGTTCATC
CAGCTGGTGCAGACCTACAACCAGCTGTTCGAGGAAAACCCCATCAACGCCAGCGGCGT
GGACGCCAAGGCCATCCTGTCTGCCAGACTGAGCAAGAGCAGACGGCTGGAAAATCTGA
TCGCCCAGCTGCCCGGCGAGAAGAAGAATGGCCTGTTCGGAAACCTGATTGCCCTGAGCC
TGGGCCTGACCCCCAACTTCAAGAGCAACTTCGACCTGGCCGAGGATGCCAAACTGCAGC
TGAGCAAGGACACCTACGACGACGACCTGGACAACCTGCTGGCCCAGATCGGCGACCAG
TACGCCGACCTGTTTCTGGCCGCCAAGAACCTGTCCGACGCCATCCTGCTGAGCGACATC
CTGAGAGTGAACACCGAGATCACCAAGGCCCCCCTGAGCGCCTCTATGATCAAGAGATA
CGACGAGCACCACCAGGACCTGACCCTGCTGAAAGCTCTCGTGCGGCAGCAGCTGCCTGA
GAAGTACAAAGAGATTTTCTTCGACCAGAGCAAGAACGGCTACGCCGGCTACATTGACG
GCGGAGCCAGCCAGGAAGAGTTCTACAAGTTCATCAAGCCCATCCTGGAAAAGATGGAC
GGCACCGAGGAACTGCTCGTGAAGCTGAACAGAGAGGACCTGCTGCGGAAGCAGCGGAC
CTTCGACAACGGCAGCATCCCCCACCAGATCCACCTGGGAGAGCTGCACGCCATTCTGCG
GCGGCAGGAAGATTTTTACCCATTCCTGAAGGACAACCGGGAAAAGATCGAGAAGATCC
TGACCTTCCGCATCCCCTACTACGTGGGCCCTCTGGCCAGGGGAAACAGCAGATTCGCCT
GGATGACCAGAAAGAGCGAGGAAACCATCACCCCCTGGAACTTCGAGGAAGTGGTGGAC
AAGGGCGCTTCCGCCCAGAGCTTCATCGAGCGGATGACCAACTTCGATAAGAACCTGCCC
AACGAGAAGGTGCTGCCCAAGCACAGCCTGCTGTACGAGTACTTCACCGTGTATAACGAG
CTGACCAAAGTGAAATACGTGACCGAGGGAATGAGAAAGCCCGCCTTCCTGAGCGGCGA
GCAGAAAAAGGCCATCGTGGACCTGCTGTTCAAGACCAACCGGAAAGTGACCGTGAAGC
AGCTGAAAGAGGACTACTTCAAGAAAATCGAGTGCTTCGACTCCGTGGAAATCTCCGGCG

TGGAAGATCGGTTCAACGCCTCCCTGGGCACATACCACGATCTGCTGAAAATTATCAAGG
ACAAGGACTTCCTGGACAATGAGGAAAACGAGGACATTCTGGAAGATATCGTGCTGACC
CTGACACTGTTTGAGGACAGAGAGATGATCGAGGAACGGCTGAAAACCTATGCCCACCT
GTTCGACGACAAAGTGATGAAGCAGCTGAAGCGGCGGAGATACACCGGCTGGGGCAGGC
TGAGCCGGAAGCTGATCAACGGCATCCGGGACAAGCAGTCCGGCAAGACAATCCTGGAT
TTCCTGAAGTCCGACGGCTTCGCCAACAGAAACTTCATGCAGCTGATCCACGACGACAGC
CTGACCTTTAAAGAGGACATCCAGAAAGCCCAGGTGTCCGGCCAGGGCGATAGCCTGCA
CGAGCACATTGCCAATCTGGCCGGCAGCCCCGCCATTAAGAAGGGCATCCTGCAGACAGT
GAAGGTGGTGGACGAGCTCGTGAAAGTGATGGGCCGGCACAAGCCCGAGAACATCGTGA
TCGAAATGGCCAGAGAGAACCAGACCACCCAGAAGGGACAGAAGAACAGCCGCGAGAG
AATGAAGCGGATCGAAGAGGGCATCAAAGAGCTGGGCAGCCAGATCCTGAAAGAACACC
CCGTGGAAAACACCCAGCTGCAGAACGAGAAGCTGTACCTGTACTACCTGCAGAATGGG
CGGGATATGTACGTGGACCAGGAACTGGACATCAACCGGCTGTCCGACTACGATGTGGA
CCATATCGTGCCTCAGAGCTTTCTGAAGGACGACTCCATCGACAACAAGGTGCTGACCAG
AAGCGACAAGAACCGGGGCAAGAGCGACAACGTGCCCTCCGAAGAGGTCGTGAAGAAG
ATGAAGAACTACTGGCGGCAGCTGCTGAACGCCAAGCTGATTACCCAGAGAAAGTTCGA
CAATCTGACCAAGGCCGAGAGAGGCGGCCTGAGCGAACTGGATAAGGCCGGCTTCATCA
AGAGACAGCTGGTGGAAACCCGGCAGATCACAAAGCACGTGGCACAGATCCTGGACTCC
CGGATGAACACTAAGTACGACGAGAATGACAAGCTGATCCGGGAAGTGAAAGTGATCAC
CCTGAAGTCCAAGCTGGTGTCCGATTTCCGGAAGGATTTCCAGTTTTACAAAGTGCGCGA
GATCAACAACTACCACCACGCCCACGACGCGTACCTGAACGCCGTCGTGGGAACCGCCCT
GATCAAAAAGTACCCTAAGCTGGAAAGCGAGTTCGTGTACGGCGACTACAAGGTGTACG
ACGTACGGAAGATGATCGCCAAGAGCGAGCAGGAAATCGGCAAGGCTACCGCCAAGTAC
TTCTTCTACAGCAACATCATGAACTTTTTCAAGACCGAGATTACCCTGGCCAACGGCGAG
ATCCGGAAGCGGCCTCTGATCGAGACAAACGGCGAAACCGGGGAGATCGTGTGGGATAA
GGGCCGGGATTTTGCCACCGTGCGGAAAGTGCTGAGCATGCCCCAAGTGAATATCGTGAA
AAAGACCGAGGTGCAGACAGGCGGCTTCAGCAAAGAGTCTATCCTGCCCAAGAGGAACA
GCGATAAGCTGATCGCCAGAAAGAAGGACTGGGACCCTAAGAAGTACGGCGGCTTCGAC
AGCCCCACCGTGGCCTATTCTGTGCTGGTGGTGGCCAAAGTGGAAAAGGGCAAGTCCAA
GAAACTGAAGAGTGTGAAAGAGCTGCTGGGGATCACCATCATGGAAAGAAGCAGCTTCG
AGAAGAATCCCATCGACTTTCTGGAAGCCAAGGGCTACAAAGAAGTGAAAAAGGACCTG
ATCATCAAGCTGCCTAAGTACTCCCTGTTCGAGCTGGAAAACGGCCGGAAGAGAATGCTG
GCCTCTGCCGGCGAACTGCAGAAGGGAAACGAACTGGCCCTGCCCTCCAAATATGTGAA
CTTCCTGTACCTGGCCAGCCACTATGAGAAGCTGAAGGGCTCCCCCGAGGATAATGAGCA
GAAACAGCTGTTTGTGGAACAGCACAAGCACTACCTGGACGAGATCATCGAGCAGATCA
GCGAGTTCTCCAAGAGAGTGATCCTGGCCGACGCTAATCTGGACAAAGTGCTGTCCGCCT
ACAACAAGCACCGGGATAAGCCCATCAGAGAGCAGGCCGAGAATATCATCCACCTGTTT

ACCCTGACCAATCTGGGAGCCCCTGCCGCCTTCAAGTACTTTGACACCACCATCGACCGG
AAGAGGTACACCAGCACCAAAGAGGTGCTGGACGCCACCCTGATCCACCAGAGCATCAC
CGGCCTGTACGAGACACGGATCGACCTGTCTCAGCTGGGAGGCGACAAAAGGCCGGCGG
CCACGAAAAAGGCCGGCCAGGCAAAAAAGAAAAAGTAAGAATTCCTAGAGCTCGCTGAT
CAGCCTCGACTGTGCCTTCTAGTTGCCAGCCATCTGTTGTTTGCCCCTCCCCCGTGCCTTCC
TTGACCCTGGAAGGTGCCACTCCCACTGTCCTTTCCTAATAAAATGAGGAAATTGCATCG
CATTGTCTGAGTAGGTGTCATTCTATTCTGGGGGGTGGGGTGGGGCAGGACAGCAAGGGG
GAGGATTGGGAAGAGAATAGCAGGCATGCTGGGGAGCGGCCGCAGGAACCCCTAGTGAT
GGAGTTGGCCACTCCCTCTCTGCGCGCTCGCTCGCTCACTGAGGCCGGGCGACCAAAGGT
CGCCCGACGCCCGGGCTTTGCCCGGGCGGCCTCAGTGAGCGAGCGAGCGCGCAGCTGCCT
GCAGGGGCGCCTGATGCGGTATTTTCTCCTTACGCATCTGTGCGGTATTTCACACCGCATA
CGTCAAAGCAACCATAGTACGCGCCCTGTAGCGGCGCATTAAGCGCGGCGGGTGTGGTG
GTTACGCGCAGCGTGACCGCTACACTTGCCAGCGCCCTAGCGCCCGCTCCTTTCGCTTTCT
TCCCTTCCTTTCTCGCCACGTTCGCCGGCTTTCCCCGTCAAGCTCTAAATCGGGGGCTCCC
TTTAGGGTTCCGATTTAGTGCTTTACGGCACCTCGACCCCAAAAAACTTGATTTGGGTGAT
GGTTCACGTAGTGGGCCATCGCCCTGATAGACGGTTTTTCGCCCTTTGACGTTGGAGTCCA
CGTTCTTTAATAGTGGACTCTTGTTCCAAACTGGAACAACACTCAACCCTATCTCGGGCTA
TTCTTTTGATTTATAAGGGATTTTGCCGATTTCGGCCTATTGGTTAAAAAATGAGCTGATT
TAACAAAAATTTAACGCGAATTTTAACAAAATATTAACGTTTACAATTTTATGGTGCACTC
TCAGTACAATCTGCTCTGATGCCGCATAGTTAAGCCAGCCCCGACACCCGCCAACACCCG
CTGACGCGCCCTGACGGGCTTGTCTGCTCCCGGCATCCGCTTACAGACAAGCTGTGACCG
TCTCCGGGAGCTGCATGTGTCAGAGGTTTTCACCGTCATCACCGAAACGCGCGAGACGAA
AGGGCCTCGTGATACGCCTATTTTTATAGGTTAATGTCATGATAATAATGGTTTCTTAGAC
GTCAGGTGGCACTTTTCGGGGAAATGTGCGCGGAACCCCTATTTGTTTATTTTTCTAAATA
CATTCAAATATGTATCCGCTCATGAGACAATAACCCTGATAAATGCTTCAATAATATTGA
AAAAGGAAGAGTATGAGTATTCAACATTTCCGTGTCGCCCTTATTCCCTTTTTTGCGGCAT
TTTGCCTTCCTGTTTTTGCTCACCCAGAAACGCTGGTGAAAGTAAAAGATGCTGAAGATC
AGTTGGGTGCACGAGTGGGTTACATCGAACTGGATCTCAACAGCGGTAAGATCCTTGAGA
GTTTTCGCCCCGAAGAACGTTTTCCAATGATGAGCACTTTTAAAGTTCTGCTATGTGGCGC
GGTATTATCCCGTATTGACGCCGGGCAAGAGCAACTCGGTCGCCGCATACACTATTCTCA
GAATGACTTGGTTGAGTACTCACCAGTCACAGAAAAGCATCTTACGGATGGCATGACAGT
AAGAGAATTATGCAGTGCTGCCATAACCATGAGTGATAACACTGCGGCCAACTTACTTCT
GACAACGATCGGAGGACCGAAGGAGCTAACCGCTTTTTTGCACAACATGGGGGATCATG
TAACTCGCCTTGATCGTTGGGAACCGGAGCTGAATGAAGCCATACCAAACGACGAGCGT
GACACCACGATGCCTGTAGCAATGGCAACAACGTTGCGCAAACTATTAACTGGCGAACTA
CTTACTCTAGCTTCCCGGCAACAATTAATAGACTGGATGGAGGCGGATAAAGTTGCAGGA
CCACTTCTGCGCTCGGCCCTTCCGGCTGGCTGGTTTATTGCTGATAAATCTGGAGCCGGTG

AGCGTGGAAGCCGCGGTATCATTGCAGCACTGGGGCCAGATGGTAAGCCCTCCCGTATCG
TAGTTATCTACACGACGGGGAGTCAGGCAACTATGGATGAACGAAATAGACAGATCGCT
GAGATAGGTGCCTCACTGATTAAGCATTGGTAACTGTCAGACCAAGTTTACTCATATATA
CTTTAGATTGATTTAAAACTTCATTTTTAATTTAAAAGGATCTAGGTGAAGATCCTTTTTG
ATAATCTCATGACCAAAATCCCTTAACGTGAGTTTTCGTTCCACTGAGCGTCAGACCCCGT
AGAAAAGATCAAAGGATCTTCTTGAGATCCTTTTTTTCTGCGCGTAATCTGCTGCTTGCAA
ACAAAAAAACCACCGCTACCAGCGGTGGTTTGTTTGCCGGATCAAGAGCTACCAACTCTT
TTTCCGAAGGTAACTGGCTTCAGCAGAGCGCAGATACCAAATACTGTCCTTCTAGTGTAG
CCGTAGTTAGGCCACCACTTCAAGAACTCTGTAGCACCGCCTACATACCTCGCTCTGCTA
ATCCTGTTACCAGTGGCTGCTGCCAGTGGCGATAAGTCGTGTCTTACCGGGTTGGACTCA
AGACGATAGTTACCGGATAAGGCGCAGCGGTCGGGCTGAACGGGGGGTTCGTGCACACA
GCCCAGCTTGGAGCGAACGACCTACACCGAACTGAGATACCTACAGCGTGAGCTATGAG
AAAGCGCCACGCTTCCCGAAGGGAGAAAGGCGGACAGGTATCCGGTAAGCGGCAGGGTC
GGAACAGGAGAGCGCACGAGGGAGCTTCCAGGGGGAAACGCCTGGTATCTTTATAGTCC
TGTCGGGTTTCGCCACCTCTGACTTGAGCGTCGATTTTTGTGATGCTCGTCAGGGGGGCGG
AGCCTATGGAAAAACGCCAGCAACGCGGCCTTTTTACGGTTCCTGGCCTTTTGCTGGCCTT
TTGCTCACATG

HypaR-SpCas9 (without sgRNA; with silent mutations) (Addgene #126757)

DNA sequence of the HypaR-SpCas9 coding cassette:
Human codon optimized *S. pyogenes* Cas9 shown in purple, NLS underlined, modified codons in red, 3xFLAG tag in *light blue*.

ATG*GACTATAAGGACCACGACGGAGACTACAAGGATCATGATATTGATTACAAAGACGATGACG*
*ATAAG*ATGGCCCCAAAGAAGAAGCGGAAGGTCGGTATCCACGGAGTCCCAGCAGCCGAC
AAGAAGTACAGCATCGGCCTGGACATCGGCACCAACTCTGTGGGCTGGGCCGTGATCACC
GACGAGTACAAGGTGCCCAGCAAGAAATTCAAGGTGCTGGGCAACACCGACCGGCACAG
CATCAAGAAGAACCTGATCGGAGCCCTGCTGTTCGACAGCGGCGAAACAGCCGAGGCCA
CCCGGCTGAAGAGAACCGCCAGAAGAAGATACACCAGACGGAAGAACCGGATCTGCTAT
CTGCAAGAGATCTTCAGCAACGAGATGGCCAAGGTGGACGACAGCTTCTTCCACAGACTG
GAAGAGTCCTTCCTGGTGGAAGAGGATAAGAAGCACGAGCGGCACCCCATCTTCGGCAA
CATCGTGGACGAGGTGGCCTACCACGAGAAGTACCCCACCATCTACCACCTGAGAAAGA
AACTGGTGGACAGCACCGACAAGGCCGACCTGCGGCTGATCTATCTGGCCCTGGCCCACA
TGATCAAGTTCCGGGGCCACTTCCTGATCGAGGGCGACCTGAACCCCGACAACAGCGACG
TGGACAAGCTGTTCATCCAGCTGGTGCAGACCTACAACCAGCTGTTCGAGGAAAACCCCA

TCAACGCCAGCGGCGTGGACGCCAAGGCCATCCTGTCTGCCAGACTGAGCAAGAGCAGA
CGGCTGGAAAATCTGATCGCCCAGCTGCCCGGCGAGAAGAAGAATGGCCTGTTCGGAAA
CCTGATTGCCCTGAGCCTGGGCCTGACCCCCAACTTCAAGAGCAACTTCGACCTGGCCGA
GGATGCCAAACTGCAGCTGAGCAAGGACACCTACGACGACGACCTGGACAACCTGCTGG
CCCAGATCGGCGACCAGTACGCCGACCTGTTTCTGGCCGCCAAGAACCTGTCCGACGCCA
TCCTGCTGAGCGACATCCTGAGAGTGAACACCGAGATCACCAAGGCCCCCCTGAGCGCCT
CTATGATCAAGAGATACGACGAGCACCACCAGGACCTGACCCTGCTGAAAGCTCTCGTGC
GGCAGCAGCTGCCTGAGAAGTACAAAGAGATTTTCTTCGACCAGAGCAAGAACGGCTAC
GCCGGCTACATTGACGGCGGAGCCAGCCAGGAAGAGTTCTACAAGTTCATCAAGCCCATC
CTGGAAAAGATGGACGGCACCGAGGAACTGCTCGTGAAGCTGAACAGAGAGGACCTGCT
GCGGAAGCAGCGGACCTTCGACAACGGCAGCATCCCCCACCAGATCCACCTGGGAGAGC
TGCACGCCATTCTGCGGCGGCAGGAAGATTTTTACCCATTCCTGAAGGACAACCGGGAAA
AGATCGAGAAGATCCTGACCTTCCGCATCCCCTACTACGTGGGCCCTCTGGCCAGGGGAA
ACAGCAGATTCGCCTGGATGACCAGAAAGAGCGAGGAAACCATCACCCCCTGGAACTTC
GAGGAAGTGGTGGACAAGGGCGCTTCCGCCCAGAGCTTCATCGAGCGGATGACCAACTT
CGATAAGAACCTGCCCAACGAGAAGGTGCTGCCCAAGCACAGCCTGCTGTACGAGTACTT
CACCGTGTATAACGAGCTGACCAAAGTGAAATACGTGACCGAGGGAATGAGAAAGCCCG
CCTTCCTGAGCGGCGAGCAGAAAAAGGCCATCGTGGACCTGCTGTTCAAGACCAACCGG
AAAGTGACCGTGAAGCAGCTGAAAGAGGACTACTTCAAGAAAATCGAGTGCTTCGACTC
CGTGGAAATCTCCGGCGTGGAAGATCGGTTCAACGCCTCCCTGGGCACATACCACGATCT
GCTGAAAATTATCAAGGACAAGGACTTCCTGGACAATGAGGAAAACGAGGACATTCTGG
AAGATATCGTGCTGACCCTGACACTGTTTGAGGACAGAGAGATGATCGAGGAACGGCTG
AAAACCTATGCCCACCTGTTCGACGACAAAGTGATGAAGCAGCTGAAGCGGCGGAGATA
CACCGGCTGGGGCGCGCTGAGCCGGAAGCTGATCAACGGCATCCGCGACAAGCAGTCCG
GCAAGACAATCCTGGATTTCCTGAAGTCCGACGGCTTCGCCAACAGAGCCTTTGCAGCCC
TGATCGCTGACGACAGCCTGACCTTTAAAGAGGACATCCAGAAAGCCCAGGTGTCCGGCC
AGGGCGATAGCCTGCACGAGCACATTGCCAATCTGGCCGGCAGCCCCGCCATTAAGAAG
GGCATCCTGCAGACAGTGAAGGTGGTGGACGAGCTCGTGAAAGTGATGGGCCGGCACAA
GCCCGAGAACATCGTGATCGAAATGGCCAGAGAGAACCAGACCACCCAGAAGGGACAG
AAGAACAGCCGCGAGAGAATGAAGCGGATCGAAGAGGGCATCAAAGAGCTGGGCAGCC
AGATCCTGAAAGAACACCCCGTGGAAAACACCCAGCTGCAGAACGAGAAGCTGTACCTG
TACTACCTGCAGAATGGGCGGGATATGTACGTGGACCAGGAACTGGACATCAACCGGCT
GTCCGACTACGATGTGGACCATATCGTGCCTCAGAGCTTTCTGAAGGACGACTCCATCGA
CAACAAGGTGCTGACCAGAAGCGACAAGAACCGGGGCAAGAGCGACAACGTGCCCTCCG
AAGAGGTCGTGAAGAAGATGAAGAACTACTGGCGGCAGCTGCTGAACGCCAAGCTGATT
ACCCAGAGAAAGTTCGACAATCTGACCAAGGCCGAGAGAGGCGGCCTGAGCGAACTGGA
TAAGGCCGGCTTCATCAAGAGACAGCTGGTGGAAACCCGGCAGATCACAAAGCACGTGG

CACAGATCCTGGACTCCCGGATGAACACTAAGTACGACGAGAATGACAAGCTGATCCGG
GAAGTGAAAGTGATCACCCTGAAGTCCAAGCTGGTGTCCGATTTCCGGAAGGATTTCCAG
TTTTACAAAGTGCGCGAGATCAACAACTACCACCACGCCCACGACGCGTACCTGAACGCC
GTCGTGGGAACCGCCCTGATCAAAAAGTACCCTAAGCTGGAAAGCGAGTTCGTGTACGG
CGACTACAAGGTGTACGACGTACGGAAGATGATCGCCAAGAGCGAGCAGGAAATCGGCA
AGGCTACCGCCAAGTACTTCTTCTACAGCAACATCATGAACTTTTTCAAGACCGAGATTA
CCCTGGCCAACGGCGAGATCCGGAAGCGGCCTCTGATCGAGACAAACGGCGAAACCGGG
GAGATCGTGTGGGATAAGGGCCGGGATTTTGCCACCGTGCGGAAAGTGCTGAGCATGCC
CCAAGTGAATATCGTGAAAAAGACCGAGGTGCAGACAGGCGGCTTCAGCAAAGAGTCTA
TCCTGCCCAAGAGGAACAGCGATAAGCTGATCGCCAGAAAGAAGGACTGGGACCCTAAG
AAGTACGGCGGCTTCGACAGCCCCACCGTGGCCTATTCTGTGCTGGTGGTGGCCAAAGTG
GAAAAGGGCAAGTCCAAGAAACTGAAGAGTGTGAAAGAGCTGCTGGGGATCACCATCAT
GGAAAGAAGCAGCTTCGAGAAGAATCCCATCGACTTTCTGGAAGCCAAGGGCTACAAAG
AAGTGAAAAAGGACCTGATCATCAAGCTGCCTAAGTACTCCCTGTTCGAGCTGGAAAACG
GCCGGAAGAGAATGCTGGCCTCTGCCGGCGAACTGCAGAAGGGAAACGAACTGGCCCTG
CCCTCCAAATATGTGAACTTCCTGTACCTGGCCAGCCACTATGAGAAGCTGAAGGGCTCC
CCCGAGGATAATGAGCAGAAACAGCTGTTTGTGGAACAGCACAAGCACTACCTGGACGA
GATCATCGAGCAGATCAGCGAGTTCTCCAAGAGAGTGATCCTGGCCGACGCTAATCTGGA
CAAAGTGCTGTCCGCCTACAACAAGCACCGGGATAAGCCCATCAGAGAGCAGGCCGAGA
ATATCATCCACCTGTTTACCCTGACCAATCTGGGAGCCCCTGCCGCCTTCAAGTACTTTGA
CACCACCATCGACCGGAAGAGGTACACCAGCACCAAAGAGGTGCTGGACGCCACCCTGA
TCCACCAGAGCATCACCGGCCTGTACGAGACACGGATCGACCTGTCTCAGCTGGGAGGCG
ACAAAAGGCCGGCGGCCACGAAAAAGGCCGGCCAGGCAAAAAAGAAAAAGTAA

pX330-xCas9 (without sgRNA) (Addgene #)

DNA sequence of the xCas9 coding cassette:

Human codon optimized *S. pyogenes* Cas9 shown in purple, NLS underlined, modified codons in red, silent mutations in yellow, 3xFLAG tag in *light blue*.

ATG*GACTATAAGGACCACGACGGAGACTACAAGGATCATGATATTGATTACAAAGACGATGACG*
*ATAAG*ATGGCCCCAAAGAAGAAGCGGAAGGTCGGTATCCACGGAGTCCCAGCAGCCGAC
AAGAAGTACAGCATCGGCCTGGACATCGGCACCAACTCTGTGGGCTGGGCCGTGATCACC
GACGAGTACAAGGTGCCCAGCAAGAAATTCAAGGTGCTGGGCAACACCGACCGGCACAG
CATCAAGAAGAACCTGATCGGAGCCCTGCTGTTCGACAGCGGCGAAACAGCCGAGGCCA
CCCGGCTGAAGAGAACCGCCAGAAGAAGATACACCAGACGGAAGAACCGGATCTGCTAT

CTGCAAGAGATCTTCAGCAACGAGATGGCCAAGGTGGACGACAGCTTCTTCCACAGACTG
GAAGAGTCCTTCCTGGTGGAAGAGGATAAGAAGCACGAGCGGCACCCCATCTTCGGCAA
CATCGTGGACGAGGTGGCCTACCACGAGAAGTACCCCACCATCTACCACCTGAGAAAGA
AACTGGTGGACAGCACCGACAAGGCCGACCTGCGGCTGATCTATCTGGCCCTGGCCCACA
TGATCAAGTTCCGGGGCCACTTCCTGATCGAGGGCGACCTGAACCCCGACAACAGCGACG
TGGACAAGCTGTTCATCCAGCTGGTGCAGACCTACAACCAGCTGTTCGAGGAAAACCCCA
TCAACGCCAGCGGCGTGGACGCCAAGGCCATCCTGTCTGCCAGACTGAGCAAGAGCAGA
CGGCTGGAAAATCTGATCGCCCAGCTGCCCGGCGAGAAGAAGAATGGCCTGTTCGGAAA
CCTGATTGCCCTGAGCCTGGGCCTGACCCCCAACTTCAAGAGCAACTTCGACCTGGCCGA
GGATACCAAACTGCAGCTGAGCAAGGACACCTACGACGACGACCTGGACAACCTGCTGG
CCCAGATCGGCGACCAGTACGCCGACCTGTTTCTGGCCGCCAAGAACCTGTCCGACGCCA
TCCTGCTGAGCGACATCCTGAGAGTGAACACCGAGATCACCAAGGCCCCCCTGAGCGCCT
CTATGATCAAGCTGTACGACGAGCACCACCAGGACCTGACCCTGCTGAAAGCTCTCGTGC
GGCAGCAGCTGCCTGAGAAGTACAAAGAGATTTTCTTCGACCAGAGCAAGAACGGCTAC
GCCGGCTACATTGACGGCGGAGCCAGCCAGGAAGAGTTCTACAAGTTCATCAAGCCCATC
CTGGAAAAGATGGACGGCACCGAGGAACTGCTCGTGAAGCTGAACAGAGAGGACCTGCT
GCGGAAGCAGCGGACCTTCGACAACGGCATCATCCCCCACCAGATCCACCTGGGAGAGC
TGCACGCCATTCTGCGGCGGCAGGAAGATTTTTACCCATTCCTGAAGGACAACCGGGAAA
AGATCGAGAAGATCCTGACCTTCCGCATCCCCTACTACGTGGGCCCTCTGGCCAGGGGAA
ACAGCAGATTCGCCTGGATGACCAGAAAGAGCGAGGAAACCATCACCCCCTGGAACTTC
GAGAAGGTGGTGGACAAGGGCGCTTCCGCCCAGAGCTTCATCGAGCGGATGACCAACTT
CGATAAGAACCTGCCCAACGAGAAGGTGCTGCCCAAGCACAGCCTGCTGTACGAGTACTT
CACCGTGTATAACGAGCTGACCAAAGTGAAATACGTGACCGAGGGAATGAGAAAGCCCG
CCTTCCTGAGCGGCGATCAGAAAAAGGCCATCGTGGACCTGCTGTTCAAGACCAACCGGA
AAGTGACCGTGAAGCAGCTGAAAGAGGACTACTTCAAGAAAATCGAGTGCTTCGACTCC
GTGGAAATCTCCGGCGTGGAAGATCGGTTCAACGCCTCCCTGGGCACATACCACGATCTG
CTGAAAATTATCAAGGACAAGGACTTCCTGGACAATGAGGAAAACGAGGACATTCTGGA
AGATATCGTGCTGACCCTGACACTGTTTGAGGACAGAGAGATGATCGAGGAACGGCTGA
AAACCTATGCCCACCTGTTCGACGACAAAGTGATGAAGCAGCTGAAGCGGCGGAGATAC
ACCGGCTGGGGCAGGCTGAGCCGGAAGCTGATCAACGGCATCCGGGACAAGCAGTCCGG
CAAGACAATCCTGGATTTCCTGAAGTCCGACGGCTTCGCCAACAGAAACTTCATCCAGCT
GATCCACGACGACAGCCTGACCTTTAAAGAGGACATCCAGAAAGCCCAGGTGTCCGGCC
AGGGCGATAGCCTGCACGAGCACATTGCCAATCTGGCCGGCAGCCCCGCCATTAAGAAG
GGCATCCTGCAGACAGTGAAGGTGGTGGACGAGCTCGTGAAAGTGATGGGCCGGCACAA
GCCCGAGAACATCGTGATCGAAATGGCCAGAGAGAACCAGACCACCCAGAAGGGACAG
AAGAACAGCCGCGAGAGAATGAAGCGGATCGAAGAGGGCATCAAAGAGCTGGGCAGCC
AGATCCTGAAAGAACACCCCGTGGAAAACACCCAGCTGCAGAACGAGAAGCTGTACCTG

TACTACCTGCAGAATGGGCGGGATATGTACGTGGACCAGGAACTGGACATCAACCGGCT
GTCCGACTACGATGTGGACCATATCGTGCCTCAGAGCTTTCTGAAGGACGACTCCATCGA
CAACAAGGTGCTGACCAGAAGCGACAAGAACCGGGGCAAGAGCGACAACGTGCCCTCCG
AAGAGGTCGTGAAGAAGATGAAGAACTACTGGCGGCAGCTGCTGAACGCCAAGCTGATT
ACCCAGAGAAAGTTCGACAATCTGACCAAGGCCGAGAGAGGCGGCCTGAGCGAACTGGA
TAAGGCCGGCTTCATCAAGAGACAGCTGGTGGAAACCCGGCAGATCACAAAGCACGTGG
CACAGATCCTGGACTCCCGGATGAACACTAAGTACGACGAGAATGACAAGCTGATCCGG
GAAGTGAAAGTGATCACCCTGAAGTCCAAGCTGGTGTCCGATTTCCGGAAGGATTTCCAG
TTTTACAAAGTGCGCGAGATCAACAACTACCACCACGCCCACGACGCGTACCTGAACGCC
GTCGTGGGAACCGCCCTGATCAAAAAGTACCCTAAGCTGGAAAGCGAGTTCGTGTACGG
CGACTACAAGGTGTACGACGTGCGGAAGATGATCGCCAAGAGCGAGCAGGAAATCGGCA
AGGCTACCGCCAAGTACTTCTTCTACAGCAACATCATGAACTTTTTCAAGACCGAGATTA
CCCTGGCCAACGGCGAGATCCGGAAGCGGCCTCTGATCGAGACAAACGGCGAAACCGGG
GAGATCGTGTGGGATAAGGGCCGGGATTTTGCCACCGTGCGGAAAGTGCTGAGCATGCC
CCAAGTGAATATCGTGAAAAAGACCGAGGTGCAGACAGGCGGCTTCAGCAAAGAGTCTA
TCCTGCCCAAGAGGAACAGCGATAAGCTGATCGCCAGAAAGAAGGACTGGGACCCTAAG
AAGTACGGCGGCTTCGACAGCCCCACCGTGGCCTATTCTGTGCTGGTGGTGGCCAAAGTG
GAAAAGGGCAAGTCCAAGAAACTGAAGAGTGTGAAAGAGCTGCTGGGGATCACCATCAT
GGAAAGAAGCAGCTTCGAGAAGAATCCCATCGACTTTCTGGAAGCCAAGGGCTACAAAG
AAGTGAAAAAGGACCTGATCATCAAGCTGCCTAAGTACTCCCTGTTCGAGCTGGAAAACG
GCCGGAAGAGAATGCTGGCCTCTGCCGGCGTGCTGCAGAAGGGAAACGAACTGGCCCTG
CCCTCCAAATATGTGAACTTCCTGTACCTGGCCAGCCACTATGAGAAGCTGAAGGGCTCC
CCCGAGGATAATGAGCAGAAACAGCTGTTTGTGGAACAGCACAAGCACTACCTGGACGA
GATCATCGAGCAGATCAGCGAGTTCTCCAAGAGAGTGATCCTGGCCGACGCTAATCTGGA
CAAAGTGCTGTCCGCCTACAACAAGCACCGGGATAAGCCCATCAGAGAGCAGGCCGAGA
ATATCATCCACCTGTTTACCCTGACCAATCTGGGAGCCCCTGCCGCCTTCAAGTACTTTGA
CACCACCATCGACCGGAAGAGGTACACCAGCACCAAAGAGGTGCTGGACGCCACCCTGA
TCCACCAGAGCATCACCGGCCTGTACGAGACACGGATCGACCTGTCTCAGCTGGGAGGCG
AC<u>AAAAGGCCGGCGGCCACGAAAAAGGCCGGCCAGGCAAAAAAGAAAAAG</u>

Supplementary Note 3. SpCas9 variants, bacterial expression plasmids

Bacterial expression plasmids [pET-FLAG-WT SpCas9 (Addgene #), pET-FLAG-SpCas9-B-HF1 (Addgene #), pET-FLAG-B-evoSpCas9 (Addgene #126773) and pET-FLAG-HeFSpCas9 (Addgene #126771)] were constructed from pMJ806 (#39312)[7] plasmid by digestion with BcuI and NotI

restriction enzymes and by ligating a fragment (containing the TEV-3xFLAG-NLS-SpCas9 variant-NLS coding sequence) to it, which was generated as follows: PCR products were generated from the mammalian expression plasmids [pX330-Flag-wtSpCas9 (without sgRNA) (Addgene #92353), B-SpCas9-HF1 (Addgene #126762), B-evoSpCas9 (Addgene #126765), pX330-Flag-HeFSpCas9 (without sgRNA; with silent mutations) (Addgene # 126759)] using the 9214-SpCas9_bact_exp-for and 9214-SpCas9_bact_exp-rev primers, and then the PCR products were digested with BcuI and NotI restriction enzymes.
Bacterial expression plasmids [pET-HypaR-SpCas9-NLS-6xHis, pET-Blackjack SpCas9-NLS-6xHis, pET-eSpCas9-plus-NLS-6xHis, pET-SpCas9-HF1-NLS-6xHis, pET-B-HypaSpCas9-NLS-6xHis, pET-B-HypaR-SpCas9-NLS-6xHis, pET-evoSpCas9-NLS-6xHis]were constructed from pET-Cas9-NLS-6xHis (Addgene #62933)[8] plasmid in two steps. In the first step, the plasmid was digested with NcoI and PacI restriction enzymes and assembled with one PCR fragment using the NEBuilder HiFi DNA Assembly Master Mix. The PCR fragment was generated from pX330-Flag-WT_SpCas9 (without sgRNA; with silent mutations) (Addgene #126753) using the 8965 Gibson-fwd and 8965 Gibson-rev primers. In the second step, the first step plasmid product was digested with BglII and Eco52I restriction enzymes and assembled with one PCR fragment using the NEBuilder HiFi DNA Assembly Master Mix. The PCR fragment was generated from the corresponding mammalian expression plasmids (Supplementary Data file 1: SpCas9 plasmids) using the 8966 Gibson-fwd and 8966 Gibson-rev primers.

Supplementary Note 4. Cloning of new B-SpCas9 variants

SpCas9 Blackjack variants were constructed from the parent SpCas9 variants (Addgene numbers: #126777, #126778, #126757) by digestion with MluI and Pfl23II restriction enzymes (cleavage sites introduced into the parent SpCas9s plasmid sequence by silent mutations). The synthetic DNA oligonucleotides (8719for and 8719rev) were hybridized, and the annealed oligonucleotides were ligated into the digested vectors.

Supplementary Note 5. Cloning of in-between increased-fidelity SpCas9 variants

B-evoSpCas9-Q661R, B-HeFSpCas9-A661R, B-HeFSpCas9-A695Q, B-HeFSpCas9-A848K and B-HeFSpCas9-A926Q variants were constructed from the parent Blackjack-SpCas9 variants (Addgene numbers: #126765, #126766) by digestion with EcoRI and EcoRV restriction enzymes and assembled with two PCR fragments using the NEBuilder HiFi DNA Assembly Master Mix. Fragment one was a PCR product generated from the parent variant using the Cas9-cloning_for and one of the following primers: A661R-rev-Gibson(B-HeF), A695Q-rev-Gibson(B-HeF), A848K-rev-Gibson(B-HeF) or

A926Q-rev-Gibson(B-HeF). Fragment two was a PCR product generated from the parent variant using the Cas9-cloning_rev and one of the following primers: A661R-fwd-Gibson(B-HeF), A695Q-fwd-Gibson(B-HeF), A848K-fwd-Gibson(B-HeF) or A926Q-fwd-Gibson(B-HeF).

B-evoSpCas9-V495M, B-evoSpCas9-N515Y, B-evoSpCas9-E526K variants were constructed from the parent Blackjack-SpCas9 variants (Addgene numbers: #126765, #126766) by digestion with EcoRI and AgeI restriction enzymes and assembled with two PCR fragments using the NEBuilder HiFi DNA Assembly Master Mix. Fragment one was a PCR product generated from the parent variant using the Cas9-cloning_for2 and one of the following primers: V495M-rev-Gibson(B-evo), N515Y-rev-Gibson(B-evo) or E526K-rev-Gibson(B-evo). Fragment two was a PCR product generated from the parent variant using the Cas9-cloning_rev and one of the following primers: V495M-fwd-Gibson(B-evo), N515Y-fwd-Gibson(B-evo) or E526K-fwd-Gibson(B-evo).

B-HeFSpCas9-A1003K was constructed from the B-HeFSpCas9 plasmid by digestion with EcoRI and MluI restriction enzymes and ligation with a fragment cleaved from the eSpCas9-plus (Addgene #126767) plasmid by digestion with EcoRI and MluI restriction enzymes.

B-HeFSpCas9-A1060R was constructed from the B-HeFSpCas9 plasmid by digestion with EcoRI and MluI restriction enzymes and ligation with a fragment cleaved from the e+3 plasmid[5] by digestion with EcoRI and MluI restriction enzymes.

Supplementary Note 6. Cloning of xCas9, SpCas9-NG and HypaR-SpCas9 variants

xCas9-L324R, xCas9-I409S, xCas9-I694M, xCas9-triple mutant, HypaR-SpCas9-L847R-V1015R, xCas9-L847R-V1015R and SpCas9-NG-L847R-V1015R variants were constructed from the parent SpCas9 variants (Addgene numbers: #, #) by digestion with BglII and EcoRI restriction enzymes and assembled with PCR fragments using the NEBuilder HiFi DNA Assembly Master Mix.

PCR fragments were generated from the parent variant as follows:

xCas9-L324R, xCas9-I409S, xCas9-I694M: Fragment one using the xcas9for-BglII and one of the following primers: xCas9mut_L324R rev, xCas9mut_I409S rev or xCas9mut_I694M rev. Fragment two using the xcas9rev-EcoRI and one of the following primers: xCas9mut_L324R for, xCas9mut_I409S for or xCas9mut_I694M for.

In case of xCas9-triple mutant: fragment one using the following primers: xcas9for-BglII, xCas9mut_L324R rev; fragment two using the following primers: xCas9mut_I409S rev, xCas9mut_L324R for; fragment three using the following primers: xCas9mut_I694M rev,

xCas9mut_I409S for; fragment four using the following primers: xcas9rev-EcoRI, xCas9mut_I694M for.

In case of HypaR-SpCas9-L847R-V1015R, xCas9-L847R-V1015R and SpCas9-NG-L847R-V1015R variants: fragment one using the following primers: xcas9for-BglII, Cas9mut_L847R-rev; fragment two using the following primers: Cas9mut_L847R-for, Cas9 mut_V1015R-rev; fragment three using the following primers: xcas9rev-EcoRI, Cas9 mut_V1015R-for.

Supplementary Note 7. Other plasmid sequences

Prnp.HA-EGFP-DHFR[DD]

1,000 bp long homology arm to the *Prnp* gene-EGFP-DHFR[DD] [EGFP-folA dihydrofolate reductase destabilization domain] fusion protein coding cassette and 1,000 bp long homology arm to the *Prnp* gene. Cloned from pBMN DHFR(DD)-YFP (#29325)[9] using standard molecular biology techniques.

Plasmid sequence:
ACATGTGAAGACAACTACTTGTGAATGCAGCTAGATGTATCTCAGCAGCCCAGCCCGCCC
GGCTCTCTTGTCTATGGAGAGAGTGAGGAATTCTCAAGTGAGCCTGCCAAAACTCTAGAT
GTTTCCTGTCTCTGATAAACTTAGGTTGAAAATTCCCTCAAGGAGATTCTTGGCTTTGTGC
TTAGGGGATGTTAATTCCGTCACTTGACAGCTGTGTTGTGTCCTCCTCTGTGCCAGGCACT
GCCCTTACCCATAAAATATGGCAACGAAACAGAGGCTCTTGGTTTGGTTTGGATTCTGGG
GCATGAGCTGTAAAGCCCAGATGTATTAGAACTCACAGCCGTCCTGTTTCAGCCTCTACTT
CCCAAGTGCTGGGGCACCAACGTGCACTTCCTCATGCCTGGCTCTGGAGACCTACTGCTT
GTCTCCAGGGCTCAAACACTGAGTCAGCTTTCTTCAAGTCCTTGCTCCTGCTGTAGCCACT
CAGGAGCCCTCCTGACTAGACCATGACTCAGGCCCTTGTGGTGTTACGGTTACTCAGGAC
CAGTGTACTCACAGCTACCCCTGCAGGTGACTTTCTGCATTCTGGGGAATGAAGCCTACA
TCCGTGGATAAAGGTTCTCCTCTGTGTAGAGGCTCACACCCCACAGGACCCTGGGGCCAT
TATAGCAGCCTTATAGTACAGCTGCCAGGCTCCCCACAAGATCATGCCCATTTCCAAATT
CCACTACATTGTTAAAGCTCAAAGCCATGGCGTAACAACCATGCAATATCACCTAGACCA
GACGTGGTTTACCAGTTGGGGTAACTCTTGTCAAATCTGTCCTCAGAGGATGGGATGAGC
TGTGTGTTTTTGATTTACTTTTTTCCTGAAGGAAAAGCTACGGGGGGGGGGGGGCGGGG
GGGAGGGTTGACGCCATGACTTTCATACATTTGCTTTGTAGATAGATGTCAAGGACCTTC
AGCCTAAATACTGGGCACTGATACCTTGTTCCTCATTTTGCAGATCAGTCATCACACCTGT
CTTCATTAATACCGGTCGCCACCATGGTGAGCAAGGGCGAGGAGCTGTTCACCGGGGTGG
TGCCCATCCTGGTCGAGCTGGACGGCGACGTAAACGGCCACAAGTTCAGCGTGTCCGGCG
AGGGCGAGGGCGATGCCACCTACGGCAAGCTGACCCTGAAGTTCATCTGCACCACCGGC

AAGCTGCCCGTGCCCTGGCCCACCCTCGTGACCACCCTGACCTACGGCGTGCAGTGCTTC
AGCCGCTACCCCGACCACATGAAGCAGCACGACTTCTTCAAGTCCGCCATGCCCGAAGGC
TACGTCCAGGAGCGCACCATCTTCTTCAAGGACGACGGCAACTACAAGACCCGCGCCGA
GGTGAAGTTCGAGGGCGACACCCTGGTGAACCGCATCGAGCTGAAGGGCATCGACTTCA
AGGAGGACGGCAACATCCTGGGGCACAAGCTGGAGTACAACTACAACAGCCACAACGTC
TATATCATGGCCGACAAGCAGAAGAACGGCATCAAGGTGAACTTCAAGATCCGCCACAA
CATCGAGGACGGCAGCGTGCAGCTCGCCGACCACTACCAGCAGAACACCCCCATCGGCG
ACGGCCCCGTGCTGCTGCCCGACAACCACTACCTGAGCACCCAGTCCGCCCTGAGCAAAG
ACCCCAACGAGAAGCGCGATCACATGGTCCTGCTGGAGTTCGTGACCGCCGCCGGGATCA
CTCTCGGCATGGACGAGCTGTACAAGGGATCCCTTGCAGAGGCGTCTGCTAAAGCGGCAG
CGGGCTCGAGTATCAGTCTGATTGCGGCGTTAGCGGTAGATTACGTTATCGGCATGGAAA
ACGCCATGCCGTGGAACCTGCCTGCCGATCTCGCCTGGTTTAAACGCAACACCTTAAATA
AACCCGTGATTATGGGCCGCCATACCTGGGAATCAATCGGTCGTCCGTTGCCAGGACGCA
AAAATATTATCCTCAGCAGTCAACCGAGTACGGACGATCGCGTAACGTGGGTGAAGTCG
GTGGATGAAGCCATCGCGGCGTGTGGTGACGTACCAGAAATCATGGTGATTGGCGGCGG
TCGCGTTATTGAACAGTTCTTGCCAAAAGCGCAAAAACTGTATCTGACGCATATCGACGC
AGAAGTGGAAGGCGACACCCATTTCCCGGATTACGAGCCGGATGACTGGGAATCGGTAT
TCAGCGAATTCCACGATGCTGATGCGCAGAACTCTCACAGCTATTGCTTTGAGATTCTGG
AGCGGCGATAGCAATTGTTGTTGTTAACTTGTTTATTGCAGCTTATAATGGTTACAAATAA
AGCAATAGCATCACAAATTTCACAAATAAAGCATTTTTTTCACTGCATTCTAGTTGTGGTT
TGTCCAAACTCATCAATGTATCTTAACGCGTCGTCTCTCCTCGGGAGGCCTTCCTGCTTGT
TCCTTCGCATTCTCGTGGTCTAGGCTGGGGGAGGGGTTATCCACCTGTAGCTCTTTCAATT
GAGGTGGTTCTCATTCTTGCTTCTCTGTGTCCCCCATAGGCTAATACCCCTGGCACTGATG
GGCCCTGGGAAATGTACAGTAGACCAGTTGCTCTTTGCTTCAGGTCCCTTTGATGGAGTCT
GTCATCAGCCAGTGCTAACACCGGGCCAATAAGAATATAACACCAAATAACTGCTGGCTA
GTTGGGGCTTTGTTTTGGTCTAGTGAATAAATACTGGTGTATCCCCTGACTTGTACCCAGA
GTACAAGGTGACAGTGACACATGTAACTTAGCATAGGCAAAGGGTTCTACAACCAAAGA
AGCCACTGTTTGGGGATGGCGCCCTGGAAAACAGCCTCCCACCTGGGATAGCTAGAGCAT
CCACACGTGGAATTCTTTCTTTACTAACAAACGATAGCTGATTGAAGGCAACAGGAAAAA
AAAAATCAAATTGTCCTACTGACGTTGAAAGCAAACCTTTGTTCATTCCCAGGGCACTAG
AATGATCTTTAGCCTTGCTTGGATTGAACTAGGAGATCTTGACTCTGAGGAGAGCCAGCC
CTGTAAAAAGCTTGGTCCTCCTGTGACGGGAGGGATGGTTAAGGTACAAAGGCTAGAAA
CTTGAGTTTCTTCATTTCTGTCTCACAATTATCAAAAGCTAGAATTAGCTTCTGCCCTATGT
TTCTGTACTTCTATTTGAACTGGATAACAGAGAGACAATCTAAACATTCTCTTAGGCTGCA
GATAAGAGAAGTAGGCTCCATTCCAAAGTGGGAAAGAAATTCTGCTAGCATTGTTTAAAT
CAGGCAAAATTTGTTCCTGAAGTTGCTTTTTACCCCAGCAGACATAAACTGCGATAGCTTC
AGCTTGCACTGTGGATTTTCTGTATAGAATATATAAAACATAACTTCAAGCTTATGTCTTC

TTTTTAAAACATCTGAAGTATGGGACGCCCTTTCTCGAGACGCACAATGTGAACCATCAC
CCTAATCAAGTTTTTTGGGGTCGAGGTGCCGTAAAGCACTAAATCGGAACCCTAAAGGGA
GCCCCCGATTTAGAGCTTGACGGGGAAAGCCGGCGAACGTGGCGAGAAAGGAAGGGAA
GAAAGCGAAAGGAGCGGGCGCTAGGGCGCTGGCAAGTGTAGCGGTCACGCTGCGCGTAA
CCACCACACCCGCCGCGCTTAATGCGCCGCTACAGGGCGCGTCAGGTGGCACTTTTCGGG
GAAATGTGCGCGGAACCCCTATTTGTTTATTTTTCTAAATACATTCAAATATGTATCCGCT
CATGAGACAATAACCCTGATAAATGCTTCAATAATATTGAAAAAGGAAGAGTCCTGAGG
CGGAAAGAACCAGCTGTGGAATGTGTGTCAGTTAGGGTGTGGAAAGTCCCCAGGCTCCCC
AGCAGGCAGAAGTATGCAAAGCATGCATCTCAATTAGTCAGCAACCAGGTGTGGAAAGT
CCCCAGGCTCCCCAGCAGGCAGAAGTATGCAAAGCATGCATCTCAATTAGTCAGCAACCA
TAGTCCCGCCCCTAACTCCGCCCATCCCGCCCCTAACTCCGCCCAGTTCCGCCCATTCTCC
GCCCCATGGCTGACTAATTTTTTTTATTTATGCAGAGGCCGAGGCCGCCTCGGCCTCTGAG
CTATTCCAGAAGTAGTGAGGAGGCTTTTTTGGAGGCCTAGGCTTTTGCAAAGATCGATCA
AGAGACAGGATGAGGATCGTTTCGCATGATTGAACAAGATGGATTGCACGCAGGTTCTCC
GGCCGCTTGGGTGGAGAGGCTATTCGGCTATGACTGGGCACAACAGACAATCGGCTGCTC
TGATGCCGCCGTGTTCCGGCTGTCAGCGCAGGGGCGCCCGGTTCTTTTTGTCAAGACCGA
CCTGTCCGGTGCCCTGAATGAACTGCAAGACGAGGCAGCGCGGCTATCGTGGCTGGCCAC
GACGGGCGTTCCTTGCGCAGCTGTGCTCGACGTTGTCACTGAAGCGGGAAGGGACTGGCT
GCTATTGGGCGAAGTGCCGGGGCAGGATCTCCTGTCATCTCACCTTGCTCCTGCCGAGAA
AGTATCCATCATGGCTGATGCAATGCGGCGGCTGCATACGCTTGATCCGGCTACCTGCCC
ATTCGACCACCAAGCGAAACATCGCATCGAGCGAGCACGTACTCGGATGGAAGCCGGTC
TTGTCGATCAGGATGATCTGGACGAAGAGCATCAGGGGCTCGCGCCAGCCGAACTGTTCG
CCAGGCTCAAGGCGAGCATGCCCGACGGCGAGGATCTCGTCGTGACCCATGGCGATGCCT
GCTTGCCGAATATCATGGTGGAAAATGGCCGCTTTTCTGGATTCATCGACTGTGGCCGGC
TGGGTGTGGCGGACCGCTATCAGGACATAGCGTTGGCTACCCGTGATATTGCTGAAGAGC
TTGGCGGCGAATGGGCTGACCGCTTCCTCGTGCTTTACGGTATCGCCGCTCCCGATTCGCA
GCGCATCGCCTTCTATCGCCTTCTTGACGAGTTCTTCTGAGCGGGACTCTGGGGTTCGAAA
TGACCGACCAAGCGACGCCCAACCTGCCATCACGAGATTTCGATTCCACCGCCGCCTTCT
ATGAAAGGTTGGGCTTCGGAATCGTTTTCCGGGACGCCGGCTGGATGATCCTCCAGCGCG
GGGATCTCATGCTGGAGTTCTTCGCCCACCCTAGGGGGAGGCTAACTGAAACACGGAAG
GAGACAATACCGGAAGGAACCCGCGCTATGACGGCAATAAAAAGACAGAATAAAACGC
ACGGTGTTGGGTCGTTTGTTCATAAACGCGGGGTTCGGTCCCAGGGCTGGCACTCTGTCG
ATACCCCACCGAGACCCCATTGGGGCCAATACGCCCGCGTTTCTTCCTTTTCCCCACCCCA
CCCCCCAAGTTCGGGTGAAGGCCCAGGGCTCGCAGCCAACGTCGGGGCGGCAGGCCCTG
CCATAGCCTCAGGTTACTCATATATACTTTAGATTGATTTAAAACTTCATTTTTAATTTAA
AAGGATCTAGGTGAAGATCCTTTTTGATAATCTCATGACCAAAATCCCTTAACGTGAGTTT
TCGTTCCACTGAGCGTCAGACCCCGTAGAAAAGATCAAAGGATCTTCTTGAGATCCTTTTT

TTCTGCGCGTAATCTGCTGCTTGCAAACAAAAAAACCACCGCTACCAGCGGTGGTTTGTT
TGCCGGATCAAGAGCTACCAACTCTTTTTCCGAAGGTAACTGGCTTCAGCAGAGCGCAGA
TACCAAATACTGTCCTTCTAGTGTAGCCGTAGTTAGGCCACCACTTCAAGAACTCTGTAGC
ACCGCCTACATACCTCGCTCTGCTAATCCTGTTACCAGTGGCTGCTGCCAGTGGCGATAA
GTCGTGTCTTACCGGGTTGGACTCAAGACGATAGTTACCGGATAAGGCGCAGCGGTCGGG
CTGAACGGGGGGTTCGTGCACACAGCCCAGCTTGGAGCGAACGACCTACACCGAACTGA
GATACCTACAGCGTGAGCTATGAGAAAGCGCCACGCTTCCCGAAGGGAGAAAGGCGGAC
AGGTATCCGGTAAGCGGCAGGGTCGGAACAGGAGAGCGCACGAGGGAGCTTCCAGGGG
GAAACGCCTGGTATCTTTATAGTCCTGTCGGGTTTCGCCACCTCTGACTTGAGCGTCGATT
TTTGTGATGCTCGTCAGGGGGGCGGAGCCTATGGAAAAACGCCAGCAACGCGGCCTTTTT
ACGGTTCCTGGCCTTTTGCTGGCCTTTTGCTC

Sprn.HA-CMV-EGFP

1,000 bp long homology arm to the *Sprn* gene-CMV-EGFP protein coding cassette and 1,000 bp long homology arm to the Shadoo (*Sprn)* gene.

Plasmid sequence:
ACATGTGAAGACAACTAACAAAATCCAGTCGTGAGCTCTGCCTAAAGAAAAGGGTCCTC
GCTGCCGCACCTTTCCGCTTGGCCGGTCAAGGCCCCTAAATCGCTATCCGACCTAGGCTTG
TGACCAGTAAGTAGGGGAGGTGGATGAACCCAGTGATGCTGGGAGGGAGGGGGAGGGG
AGAGAGGACCTGAGGAGGATGGAGCTGCCGCCACCGAGATGGCTGGTCCACAGCCAGCC
GGAACCCATCCTGATCGGTTTACCTGTCCAGGATACTGCCCTGGACAAACCCAAAAAGGG
TGGAGCTGGAGCGGGGAAGAGACGTAATTACCTAGGGTCTGGGCCTGCTGGGCGGTTCA
CCCATCGCTAGTTGTTTGCGTTGCAAGGAATCGCTGATCTGGATTCTGACCCCCACCCTCA
CCCAGTGCATTCAGCCGCAGCCACTGGCTGAAAGACTATCTCTTAAGGCATCAGGAGATC
CAGATGCCAGAGCAAAAGTCACAAGGCTGTCCATTCTAACCATAATCTGGGGGTATTGAA
GGCTCTCCATTCCAAACTAGAATCCTAATCCACTAATCCATCCCCTTCCAGAGACTCGTGT
GCAGAGCGGGGGATTTGTGCCCCCCCCCCAGGCCTGAGCCCCACTGTAGGAGCTCCGCAA
ACCCCATTCTGGGACCCATCTCCACCCTATCACAATAGTAAAACGGCCGCCCTGTAGTTA
AACCCTTTCCCCACCACCCCATCTCCACCTGGTTATACAGCAGGAACCCAAAACCAAAGT
CCTGGTGCTGGAGTTTAAAGAACCCCTTCCCCAACCCTGAGCCCACTGTCCTCCAAGATG
CTGGGAGCCCTCTATCGGATTACCACAAAACCAGAGGCTGAAGTAGGTGTCCCCAGGTCC
AGATGAGCCTATTCCCAAGCCCTGATACCCTCTTGCCCTGGTCCTAAACCACGCTCCACCC
CTGCACAGAAGCTGAAGCCCCTTCCACCCTCTTCTCGCAGATTCTGCCCAGTAGGACACC
TGTCTTCATTAATAGTAATCAATTACGGGGTCATTAGTTCATAGCCCATATATGGAGTTCC
GCGTTACATAACTTACGGTAAATGGCCCGCCTGGCTGACCGCCCAACGACCCCCGCCCAT

TGACGTCAATAATGACGTATGTTCCCATAGTAACGCCAATAGGGACTTTCCATTGACGTC
AATGGGTGGAGTATTTACGGTAAACTGCCCACTTGGCAGTACATCAAGTGTATCATATGC
CAAGTACGCCCCCTATTGACGTCAATGACGGTAAATGGCCCGCCTGGCATTATGCCCAGT
ACATGACCTTATGGGACTTTCCTACTTGGCAGTACATCTACGTATTAGTCATCGCTATTAC
CATGGTGATGCGGTTTTGGCAGTACATCAATGGGCGTGGATAGCGGTTTGACTCACGGGG
ATTTCCAAGTCTCCACCCCATTGACGTCAATGGGAGTTTGTTTTGGCACCAAAATCAACG
GGACTTTCCAAAATGTCGTAACAACTCCGCCCCATTGACGCAAATGGGCGGTAGGCGTGT
ACGGTGGGAGGTCTATATAAGCAGAGCTGGTTTAGTGAACCGTCAGATCCGCTAGCGCTA
CCGGTCGCCACCATGGTGAGCAAGGGCGAGGAGCTGTTCACCGGGGTGGTGCCCATCCTG
GTCGAGCTGGACGGCGACGTAAACGGCCACAAGTTCAGCGTGTCCGGCGAGGGCGAGGG
CGATGCCACCTACGGCAAGCTGACCCTGAAGTTCATCTGCACCACCGGCAAGCTGCCCGT
GCCCTGGCCCACCCTCGTGACCACCCTGACCTACGGCGTGCAGTGCTTCAGCCGCTACCC
CGACCACATGAAGCAGCACGACTTCTTCAAGTCCGCCATGCCCGAAGGCTACGTCCAGGA
GCGCACCATCTTCTTCAAGGACGACGGCAACTACAAGACCCGCGCCGAGGTGAAGTTCG
AGGGCGACACCCTGGTGAACCGCATCGAGCTGAAGGGCATCGACTTCAAGGAGGACGGC
AACATCCTGGGGCACAAGCTGGAGTACAACTACAACAGCCACAACGTCTATATCATGGCC
GACAAGCAGAAGAACGGCATCAAGGTGAACTTCAAGATCCGCCACAACATCGAGGACGG
CAGCGTGCAGCTCGCCGACCACTACCAGCAGAACACCCCCATCGGCGACGGCCCCGTGCT
GCTGCCCGACAACCACTACCTGAGCACCCAGTCCGCCCTGAGCAAAGACCCCAACGAGA
AGCGCGATCACATGGTCCTGCTGGAGTTCGTGACCGCCGCCGGGATCACTCTCGGCATGG
ACGAGCTGTACAAGTCCGGACTCAGATCTCGAGCTCAAGCTTCGAATTCTGCAGTCGACG
GTACCGCGGGCCCGGGATCCACCGGATCTAGATAACTGATCATAATCAGCCATACCACAT
TTGTAGAGGTTTTACTTGCTTTAAAAAACCTCCCACACCTCCCCCTGAACCTGAAACATAA
AATGAATGCAATTGTTGTTGTTAACTTGTTTATTGCAGCTTATAATGGTTACAAATAAAGC
AATAGCATCACAAATTTCACAAATAAAGCATTTTTTTCACTGCATTCTAGTTGTGGTTTGT
CCAAACTCATCAATGTATCTTAACGCGTCGTCTCTCCTCACCAGGCTAAACTCCATCCCAG
GTCTAGCTCCTAGCCTGTCTTAAGGCCCCTAGGGCCCACCCTAATGGCCTCCTGCCCAGG
GGGTAACTTCATTTTGCTAACTATGATTCCCCAGCCCAGAGAAGAGTCTGGCCATTCTGG
GCCCACAAGGCCTCTTGCAATTTGTGGATGTAGTTTACTCTCTTGCCCATCCCAGTTTCCC
AGTCTTCCACTCTGGCAGGAACTAACAGCCCTACTACCAGAGAGGCAGGCTGCCCTTGAC
TCTCCAGAACTGCCCAAGCAGGCATGCCTGTCTGCTTGCCCACCCTGACACGAGGACATG
AACAATGCTGACCGCCAAGAAGAGGCCATCCTTGGGTGGGCCCGTCTGATTCTGCCACTA
AAGCCCCCAAAGACCTTGAGCCTCCCATGGAACCAGAGGGACTTATAGTTGATGAAGAG
AGCATTCACTTAGACTGCAGTGTGAGAAATGTCAGGTGCTAACCACCTGCCCAGAGCAGG
TCCCAATGATGTCATAGCTCAGAAAACATCCAGAGCAGTAAAATAACCATCCCAGGACG
CCCACCATGCTCCTCCTACAACATGCTACCAAAGCCAAGTAGTATGTTTCTTCCTGGCAGA
CTGCCTAAGAGACCTTGTGTCAGAAGGGTTTCCACTTGAAGCCACTTGGTCCTAAAATCC

ACTGAGGGTAGAGGTTTTGAATACACTTTCAAAAACATTCCATTCTGCTTGAGCTTAAGG
GTCATGAGTGAGGGTCACTTGGATATAATACCAATCCTGCTGGGGCCTTCTTTGTATATAA
CCCAAACTGCAAGATTCCCATAGTTCCAGTAGATAGCAGCATTTTATGTTGGGAGACCCC
TCCCTTGGAAACGGTTGGACGGGGTTGGGGTGGGGAGGTGAGACAGAGCATGGCACAGC
TGACAGCTGGCAAACTGAACAGTGGAAGGGGCAGCAGATCTACAGCCCCACTGTGCCAG
AACAAAGCTAGCAGACAGATTCTCGAGACGCACTACGTGAACCATCACCCTAATCAAGTT
TTTTGGGGTCGAGGTGCCGTAAAGCACTAAATCGGAACCCTAAAGGGAGCCCCCGATTTA
GAGCTTGACGGGGAAAGCCGGCGAACGTGGCGAGAAAGGAAGGGAAGAAAGCGAAAGG
AGCGGGCGCTAGGGCGCTGGCAAGTGTAGCGGTCACGCTGCGCGTAACCACCACACCCG
CCGCGCTTAATGCGCCGCTACAGGGCGCGTCAGGTGGCACTTTTCGGGGAAATGTGCGCG
GAACCCCTATTTGTTTATTTTTCTAAATACATTCAAATATGTATCCGCTCATGAGACAATA
ACCCTGATAAATGCTTCAATAATATTGAAAAAGGAAGAGTCCTGAGGCGGAAAGAACCA
GCTGTGGAATGTGTGTCAGTTAGGGTGTGGAAAGTCCCCAGGCTCCCCAGCAGGCAGAA
GTATGCAAAGCATGCATCTCAATTAGTCAGCAACCAGGTGTGGAAAGTCCCCAGGCTCCC
CAGCAGGCAGAAGTATGCAAAGCATGCATCTCAATTAGTCAGCAACCATAGTCCCGCCCC
TAACTCCGCCCATCCCGCCCCTAACTCCGCCCAGTTCCGCCCATTCTCCGCCCCATGGCTG
ACTAATTTTTTTTATTTATGCAGAGGCCGAGGCCGCCTCGGCCTCTGAGCTATTCCAGAAG
TAGTGAGGAGGCTTTTTTGGAGGCCTAGGCTTTTGCAAAGATCGATCAAGAGACAGGATG
AGGATCGTTTCGCATGATTGAACAAGATGGATTGCACGCAGGTTCTCCGGCCGCTTGGGT
GGAGAGGCTATTCGGCTATGACTGGGCACAACAGACAATCGGCTGCTCTGATGCCGCCGT
GTTCCGGCTGTCAGCGCAGGGGCGCCCGGTTCTTTTTGTCAAGACCGACCTGTCCGGTGC
CCTGAATGAACTGCAAGACGAGGCAGCGCGGCTATCGTGGCTGGCCACGACGGGCGTTC
CTTGCGCAGCTGTGCTCGACGTTGTCACTGAAGCGGGAAGGGACTGGCTGCTATTGGGCG
AAGTGCCGGGGCAGGATCTCCTGTCATCTCACCTTGCTCCTGCCGAGAAAGTATCCATCA
TGGCTGATGCAATGCGGCGGCTGCATACGCTTGATCCGGCTACCTGCCCATTCGACCACC
AAGCGAAACATCGCATCGAGCGAGCACGTACTCGGATGGAAGCCGGTCTTGTCGATCAG
GATGATCTGGACGAAGAGCATCAGGGGCTCGCGCCAGCCGAACTGTTCGCCAGGCTCAA
GGCGAGCATGCCCGACGGCGAGGATCTCGTCGTGACCCATGGCGATGCCTGCTTGCCGAA
TATCATGGTGGAAAATGGCCGCTTTTCTGGATTCATCGACTGTGGCCGGCTGGGTGTGGC
GGACCGCTATCAGGACATAGCGTTGGCTACCCGTGATATTGCTGAAGAGCTTGGCGGCGA
ATGGGCTGACCGCTTCCTCGTGCTTTACGGTATCGCCGCTCCCGATTCGCAGCGCATCGCC
TTCTATCGCCTTCTTGACGAGTTCTTCTGAGCGGGACTCTGGGGTTCGAAATGACCGACCA
AGCGACGCCCAACCTGCCATCACGAGATTTCGATTCCACCGCCGCCTTCTATGAAAGGTT
GGGCTTCGGAATCGTTTTCCGGGACGCCGGCTGGATGATCCTCCAGCGCGGGGATCTCAT
GCTGGAGTTCTTCGCCCACCCTAGGGGGAGGCTAACTGAAACACGGAAGGAGACAATAC
CGGAAGGAACCCGCGCTATGACGGCAATAAAAAGACAGAATAAAACGCACGGTGTTGGG
TCGTTTGTTCATAAACGCGGGGTTCGGTCCCAGGGCTGGCACTCTGTCGATACCCCACCG

AGACCCCATTGGGGCCAATACGCCCGCGTTTCTTCCTTTTCCCCACCCCACCCCCCAAGTT
CGGGTGAAGGCCCAGGGCTCGCAGCCAACGTCGGGGCGGCAGGCCCTGCCATAGCCTCA
GGTTACTCATATATACTTTAGATTGATTTAAAACTTCATTTTTAATTTAAAAGGATCTAGG
TGAAGATCCTTTTTGATAATCTCATGACCAAAATCCCTTAACGTGAGTTTTCGTTCCACTG
AGCGTCAGACCCCGTAGAAAAGATCAAAGGATCTTCTTGAGATCCTTTTTTTCTGCGCGT
AATCTGCTGCTTGCAAACAAAAAAACCACCGCTACCAGCGGTGGTTTGTTTGCCGGATCA
AGAGCTACCAACTCTTTTTCCGAAGGTAACTGGCTTCAGCAGAGCGCAGATACCAAATAC
TGTTCTTCTAGTGTAGCCGTAGTTAGGCCACCACTTCAAGAACTCTGTAGCACCGCCTACA
TACCTCGCTCTGCTAATCCTGTTACCAGTGGCTGCTGCCAGTGGCGATAAGTCGTGTCTTA
CCGGGTTGGACTCAAGACGATAGTTACCGGATAAGGCGCAGCGGTCGGGCTGAACGGGG
GGTTCGTGCACACAGCCCAGCTTGGAGCGAACGACCTACACCGAACTGAGATACCTACA
GCGTGAGCTATGAGAAAGCGCCACGCTTCCCGAAGGGAGAAAGGCGGACAGGTATCCGG
TAAGCGGCAGGGTCGGAACAGGAGAGCGCACGAGGGAGCTTCCAGGGGGAAACGCCTG
GTATCTTTATAGTCCTGTCGGGTTTCGCCACCTCTGACTTGAGCGTCGATTTTTGTGATGCT
CGTCAGGGGGGCGGAGCCTATGGAAAAACGCCAGCAACGCGGCCTTTTTACGGTTCCTGG
CCTTTTGCTGGCCTTTTGCTC

‘Self-cleaving’ EGFP-expression plasmid[10]

Plasmid contains an EGFP coding cassette (without promoter), an EF1α promoter-mCherry cassette, and it harbours a genomic-targetless sgRNA as well as its target sequence (in case of perfect NHEJ integration the EGFP coding cassette will be in-frame with the genomic sequence).

Plasmid sequence:
TTTTTACGGTTCCTGGCCTTTTGCTGGCCTTTTGCTCACATGTGACACAGATCAATTTCGCC
GCAACGCCGGATACTAGTGCTGAGGCAGCCGCAAAAGAGGCAGCCGCGAAGGCACCGGT
CGCCACCATGGTGAGCAAGGGCGAGGAGCTGTTCACCGGGGTGGTGCCCATCCTGGTCG
AGCTGGACGGCGACGTAAACGGCCACAAGTTCAGCGTGTCCGGCGAGGGCGAGGGCGAT
GCCACCTACGGCAAGCTGACCCTGAAGTTCATCTGCACCACCGGCAAGCTGCCCGTGCCC
TGGCCCACCCTCGTGACCACCCTGACCTACGGCGTGCAGTGCTTCAGCCGCTACCCCGAC
CACATGAAGCAGCACGACTTCTTCAAGTCCGCCATGCCCGAAGGCTACGTCCAGGAGCGC
ACCATCTTCTTCAAGGACGACGGCAACTACAAGACCCGCGCCGAGGTGAAGTTCGAGGG
CGACACCCTGGTGAACCGCATCGAGCTGAAGGGCATCGACTTCAAGGAGGACGGCAACA
TCCTGGGGCACAAGCTGGAGTACAACTACAACAGCCACAACGTCTATATCATGGCCGACA
AGCAGAAGAACGGCATCAAGGTGAACTTCAAGATCCGCCACAACATCGAGGACGGCAGC
GTGCAGCTCGCCGACCACTACCAGCAGAACACCCCCATCGGCGACGGCCCCGTGCTGCTG
CCCGACAACCACTACCTGAGCACCCAGTCCGCCCTGAGCAAAGACCCCAACGAGAAGCG

CGATCACATGGTCCTGCTGGAGTTCGTGACCGCCGCCGGGATCACTCTCGGCATGGACGA
GCTGTACAAGTCCGGACTCAGATCTCGAGAAGCTTGATCGCGTCGTGAGGCTCCGATGCC
CGTCAGTGGGCAGAGCGCACATCGCCCACAGTCCCCGAGAAGTTGGGGGGAGGGGTCGG
CAATTGAACCGGTGCCTAGAGAAGGTGGCGCGGGGTAAACTGGGAAAGTGATGTCGTGT
ACTGGCTCCGCCTTTTTCCCGAGGGTGGGGGAGAACCGTATATAAGTGCAGTAGTCGCCG
TGAACGTTCTTTTTCGCAACGGGTTTGCCGCCAGAACACAGGATCCGTTTAAACAGCTAG
CTTCTGCAGCACGTGTCTAGAATGGTGAGCAAGGGCGAGGAGGATAACATGGCCATCAT
CAAGGAGTTCATGCGCTTCAAGGTGCACATGGAGGGCTCCGTGAACGGCCACGAGTTCG
AGATCGAGGGCGAGGGCGAGGGCCGCCCCTACGAGGGCACCCAGACCGCCAAGCTGAAG
GTGACCAAGGGTGGCCCCCTGCCCTTCGCCTGGGACATCCTGTCCCCTCAGTTCATGTACG
GCTCCAAGGCCTACGTGAAGCACCCCGCCGACATCCCCGACTACTTGAAGCTGTCCTTCC
CCGAGGGCTTCAAGTGGGAGCGCGTGATGAACTTCGAGGACGGCGGCGTGGTGACCGTG
ACCCAGGACTCCTCCCTGCAGGACAGCGAGTTCATCTACAAGGTGAAGCTGCGCGGCACC
AACTTCCCCTCCGACGGCCCCGTAATGCAGAAGAAGACCATGGGCTGGGAGGCCTCCTCC
GAGCGGATGTACCCCGAGGACGGCGCCCTGAAGGGCGAGATCAAGCAGAGGCTGAAGCT
GAAGGACGGCGGCCACTACGACGCTGAGGTCAAGACCACCTACAAGGCCAAGAAGCCCG
TGCAGCTGCCCGGCGCCTACAACGTCAACATCAAGTTGGACATCACCTCCCACAACGAGG
ACTACACCATCGTGGAACAGTACGAACGCGCCGAGGGCCGCCACTCCACCGGCGGCATG
GACGAGCTGTACAAGGTGGTACCGCGGGCCCGGGATCCACCGGATCTAGATAACTGATC
ATAATCAGCCATACCACATTTGTAGAGGTTTTACTTGCTTTAAAAAACCTCCCACACCTCC
CCCTGAACCTGAAACATAAAATGAATGCAATTGTTGTTGTTAACTTGTTTATTGCAGCTTA
TAATGGTTACAAATAAAGCAATAGCATCACAAATTTCACAAATAAAGCATTTTTTTCACT
GCATTCTAGTTGTGGTTTGTCCAAACTCATCAATGTATCTTAACGCGTAAATTGTAAGCGT
TAATATTTTGTTAAAATTCGCGTTAAATTTTTGTTAAATCAGCTCATTTTTTAACCAATAG
GCCGAAATCGGCAAAATCCCTTATAAATCAAAAGAATAGACCGAGATAGGGTTGAGTGT
TGTTCCAGTTTGGAACAAGAGTCCACTATTAAAGAACGTGGACTCCAACGTCAAAGGGCG
AAAAACCGTCTATCAGGGCGATGGCCCACTACGTGAACCATCACCCTAATCAAGTTTTTT
GGGGTCGAGGTGCCGTAAAGCACTAAATCGGAACCCTAAAGGGAGCCCCCGATTTAGAG
CTTGACGGGGAAAGCCGGCGAACGTGGCGAGAAAGGAAGGGAAGAAAGCGAAAGGAGC
GGGCGCTAGGGCGCTGGCAAGTGTAGCGGTCACGCTGCGCGTAACCACCACACCCGCCG
CGCTTAATGCGCCGCTACAGGGCGCGTCAGGTGGCACTTTTCGGGGAAATGTGCGCGGAA
CCCCTATTTGTTTATTTTTCTAAATACATTCAAATATGTATCCGCTCATGAGACAATAACC
CTGATAAATGCTTCAATAATATTGAAAAAGGAAGAGTCCTGAGGCGGAAAGAACCAGCT
GTGGAATGTGTGTCAGTTAGGGTGTGGAAAGTCCCCAGGCTCCCCAGCAGGCAGAAGTAT
GCAAAGCATGCATCTCAATTAGTCAGCAACCAGGTGTGGAAAGTCCCCAGGCTCCCCAGC
AGGCAGAAGTATGCAAAGCATGCATCTCAATTAGTCAGCAACCATAGTCCCGCCCCTAAC
TCCGCCCATCCCGCCCCTAACTCCGCCCAGTTCCGCCCATTCTCCGCCCCATGGCTGACTA

ATTTTTTTTATTTATGCAGAGGCCGAGGCCGCCTCGGCCTCTGAGCTATTCCAGAAGTAGT
GAGGAGGCTTTTTTGGAGGCCTAGGCTTTTGCAAAGATCGATCAAGAGACAGGATGAGG
ATCGTTTCGCATGATTGAACAAGATGGATTGCACGCAGGTTCTCCGGCCGCTTGGGTGGA
GAGGCTATTCGGCTATGACTGGGCACAACAGACAATCGGCTGCTCTGATGCCGCCGTGTT
CCGGCTGTCAGCGCAGGGGCGCCCGGTTCTTTTTGTCAAGACCGACCTGTCCGGTGCCCT
GAATGAACTGCAAGACGAGGCAGCGCGGCTATCGTGGCTGGCCACGACGGGCGTTCCTT
GCGCAGCTGTGCTCGACGTTGTCACTGAAGCGGGAAGGGACTGGCTGCTATTGGGCGAA
GTGCCGGGGCAGGATCTCCTGTCATCTCACCTTGCTCCTGCCGAGAAAGTATCCATCATG
GCTGATGCAATGCGGCGGCTGCATACGCTTGATCCGGCTACCTGCCCATTCGACCACCAA
GCGAAACATCGCATCGAGCGAGCACGTACTCGGATGGAAGCCGGTCTTGTCGATCAGGA
TGATCTGGACGAAGAGCATCAGGGGCTCGCGCCAGCCGAACTGTTCGCCAGGCTCAAGG
CGAGCATGCCCGACGGCGAGGATCTCGTCGTGACCCATGGCGATGCCTGCTTGCCGAATA
TCATGGTGGAAAATGGCCGCTTTTCTGGATTCATCGACTGTGGCCGGCTGGGTGTGGCGG
ACCGCTATCAGGACATAGCGTTGGCTACCCGTGATATTGCTGAAGAGCTTGGCGGCGAAT
GGGCTGACCGCTTCCTCGTGCTTTACGGTATCGCCGCTCCCGATTCGCAGCGCATCGCCTT
CTATCGCCTTCTTGACGAGTTCTTCTGAGCGGGACTCTGGGGTTCGAAATGACCGACCAA
GCGACGCCCAACCTGCCATCACGAGATTTCGATTCCACCGCCGCCTTCTATGAAAGGTTG
GGCTTCGGAATCGTTTTCCGGGACGCCGGCTGGATGATCCTCCAGCGCGGGGATCTCATG
CTGGAGTTCTTCGCCCACCCTAGGGGGAGGCTAACTGAAACACGGAAGGAGACAATACC
GGAAGGAACCCGCGCTATGACGGCAATAAAAAGACAGAATAAAACGCACGGTGTTGGGT
CGTTTGTTCATAAACGCGGGGTTCGGTCCCAGGGCTGGCACTCTGTCGATACCCCACGCC
TATTTCCCATGATTCCTTCATATTTGCATATACGATACAAGGCTGTTAGAGAGATAATTGG
AATTAATTTGACTGTAAACACAAAGATATTAGTACAAAATACGTGACGTAGAAAGTAATA
ATTTCTTGGGTAGTTTGCAGTTTTAAAATTATGTTTTAAAATGGACTATCATATGCTTACC
GTAACTTGAAAGTATTTCGATTTCTTGGCTTTATATATCTTGTGGAAAGGACGAAACACCG
ATCAATTTCGCCGCAACGCGTTTTAGAGCTAGAAATAGCAAGTTAAAATAAGGCTAGTCC
GTTATCAACTTGAAAAAGTGGCACCGAGTCGGTGCTTTTTTCCACCGAGACCCCATTGGG
GCCAATACGCCCGCGTTTCTTCCTTTTCCCCACCCCACCCCCCAAGTTCGGGTGAAGGCCC
AGGGCTCGCAGCCAACGTCGGGGCGGCAGGCCCTGCCATAGCCTCAGGTTACTCATATAT
ACTTTAGATTGATTTAAAACTTCATTTTTAATTTAAAAGGATCTAGGTGAAGATCCTTTTT
GATAATCTCATGACCAAAATCCCTTAACGTGAGTTTTCGTTCCACTGAGCGTCAGACCCC
GTAGAAAAGATCAAAGGATCTTCTTGAGATCCTTTTTTTCTGCGCGTAATCTGCTGCTTGC
AAACAAAAAAACCACCGCTACCAGCGGTGGTTTGTTTGCCGGATCAAGAGCTACCAACTC
TTTTTCCGAAGGTAACTGGCTTCAGCAGAGCGCAGATACCAAATACTGTTCTTCTAGTGTA
GCCGTAGTTAGGCCACCACTTCAAGAACTCTGTAGCACCGCCTACATACCTCGCTCTGCT
AATCCTGTTACCAGTGGCTGCTGCCAGTGGCGATAAGTCGTGTCTTACCGGGTTGGACTC
AAGACGATAGTTACCGGATAAGGCGCAGCGGTCGGGCTGAACGGGGGGTTCGTGCACAC

AGCCCAGCTTGGAGCGAACGACCTACACCGAACTGAGATACCTACAGCGTGAGCTATGA
GAAAGCGCCACGCTTCCCGAAGGGAGAAAGGCGGACAGGTATCCGGTAAGCGGCAGGGT
CGGAACAGGAGAGCGCACGAGGGAGCTTCCAGGGGGAAACGCCTGGTATCTTTATAGTC
CTGTCGGGTTTCGCCACCTCTGACTTGAGCGTCGATTTTTGTGATGCTCGTCAGGGGGGCG
GAGCCTATGGAAAAACGCCAGCAACGCGGCC

Supplementary References

1 Kulcsar, P. I. *et al.* Crossing enhanced and high fidelity SpCas9 nucleases to optimize specificity and cleavage. *Genome Biol* **18**, 190, doi:10.1186/s13059-017-1318-8 (2017).

2 Rose, J. C. *et al.* Suppression of unwanted CRISPR-Cas9 editing by co-administration of catalytically inactivating truncated guide RNAs. *Nat Commun* **11**, 2697, doi:10.1038/s41467-020-16542-9 (2020).

3 Kim, N. *et al.* Prediction of the sequence-specific cleavage activity of Cas9 variants. *Nat Biotechnol* **38**, 1328-1336, doi:10.1038/s41587-020-0537-9 (2020).

4 Bae, S., Park, J. & Kim, J. S. Cas-OFFinder: a fast and versatile algorithm that searches for potential off-target sites of Cas9 RNA-guided endonucleases. *Bioinformatics* **30**, 1473-1475, doi:10.1093/bioinformatics/btu048 (2014).

5 Kulcsar, P. I. *et al.* Blackjack mutations improve the on-target activities of increased fidelity variants of SpCas9 with 5'G-extended sgRNAs. *Nat Commun* **11**, 1223, doi:10.1038/s41467-020-15021-5 (2020).

6 Engler, C., Kandzia, R. & Marillonnet, S. A One Pot, One Step, Precision Cloning Method with High Throughput Capability. *Plos One* **3**, doi:ARTN e3647

10.1371/journal.pone.0003647 (2008).

7 Jinek, M. *et al.* A programmable dual-RNA-guided DNA endonuclease in adaptive bacterial immunity. *Science* **337**, 816-821, doi:10.1126/science.1225829 (2012).

8 Zuris, J. A. *et al.* Cationic lipid-mediated delivery of proteins enables efficient protein-based genome editing in vitro and in vivo. *Nat Biotechnol* **33**, 73-80, doi:10.1038/nbt.3081 (2015).

9 Iwamoto, M., Bjorklund, T., Lundberg, C., Kirik, D. & Wandless, T. J. A general chemical method to regulate protein stability in the mammalian central nervous system. *Chem Biol* **17**, 981-988, doi:10.1016/j.chembiol.2010.07.009 (2010).

10 Talas, A. *et al.* A convenient method to pre-screen candidate guide RNAs for CRISPR/Cas9 gene editing by NHEJ-mediated integration of a 'self-cleaving' GFP-expression plasmid. *DNA Res* **24**, 609-621, doi:10.1093/dnares/dsx029 (2017).
