# Supplementary material for: A cleavage rule for selection of increased-fidelity SpCas9 variants with high efficiency and no detectable off-targets
Source: Nat Commun. 2023 Sep 16;14:5746. doi: 10.1038/s41467-023-41393-5 (PMC10505190; doi:10.1038/s41467-023-41393-5)
Supplement: Supplementary file 2 — Reporting Summary [file 41467_2023_41393_MOESM2_ESM.pdf]

nature portfolio

Corresponding author(s): Ervin Welker

Last updated by author(s): Aug 18, 2023

Reporting Summary

Nature Portfolio wishes to improve the reproducibility of the work that we publish. This form provides structure for consistency and transparency in reporting. For further information on Nature Portfolio policies, see our Editorial Policies and the Editorial Policy Checklist.

Statistics

For all statistical analyses, confirm that the following items are present in the figure legend, table legend, main text, or Methods section.

n/a	Confirmed	
☐	☒	The exact sample size (*n*) for each experimental group/condition, given as a discrete number and unit of measurement
☐	☒	A statement on whether measurements were taken from distinct samples or whether the same sample was measured repeatedly
☐	☒	The statistical test(s) used AND whether they are one- or two-sided<br>*Only common tests should be described solely by name; describe more complex techniques in the Methods section.*
☒	☐	A description of all covariates tested
☐	☒	A description of any assumptions or corrections, such as tests of normality and adjustment for multiple comparisons
☐	☒	A full description of the statistical parameters including central tendency (e.g. means) or other basic estimates (e.g. regression coefficient) AND variation (e.g. standard deviation) or associated estimates of uncertainty (e.g. confidence intervals)
☐	☒	For null hypothesis testing, the test statistic (e.g. *F*, *t*, *r*) with confidence intervals, effect sizes, degrees of freedom and *P* value noted<br>*Give P values as exact values whenever suitable.*
☒	☐	For Bayesian analysis, information on the choice of priors and Markov chain Monte Carlo settings
☒	☐	For hierarchical and complex designs, identification of the appropriate level for tests and full reporting of outcomes
☒	☐	Estimates of effect sizes (e.g. Cohen's *d*, Pearson's *r*), indicating how they were calculated

Our web collection on statistics for biologists contains articles on many of the points above.

Software and code

Policy information about availability of computer code

Data collection	Flow cytometry and data collection was carried out using an Attune NxT Acoustic Focusing Cytometer (Applied Biosystems by Life Technologies) and Attune NxT Software v.2.7.0 was used for data analysis. Samples were sequenced on NextSeq (Illumina) with paired-end sequencing resulting in 2 ×150 bp reads, by Deltabio Ltd.
Data analysis	For flow cytometry data analysis Attune NxT Software v.2.7.0 was used.<br>GUIDE-seq data were analysed using open-source guideseq software (version 1.1). Consolidated reads were mapped to the human reference genome GrCh37 supplemented with the integrated EGFP sequence<br>Indel analysis by next-generation sequencing (NGS): Reads were aligned to the reference sequence using BBMap. Indels were counted computationally amongst the aligned reads that matched at least 75% of the first 20bp of the reference amplicon. Indels without mismatches were searched starting at ±2 bp around the cut site. For each sample, the indel frequency was determined as (number of reads with an indel) / (number of total reads). The 15 bp long centre fragment of the GUIDE-seq dsODN sequence (“gttgtcatatgttaa” / “ttaacatatgacaac”) was counted in the aligned reads to measure dsODN on-target tag integration for GUIDE-seq experiments. The ssDNA repair was determined as (number of reads with desired edit) / (number of total reads). Results can be found in Supplementary Data file 2. The following software were used: BBMap 38.08, samtools 1.8, BioPython 1.71, PySam 0.13.<br>Statistical analyses were performed using GraphPad Prism 9.

For manuscripts utilizing custom algorithms or software that are central to the research but not yet described in published literature, software must be made available to editors and reviewers. We strongly encourage code deposition in a community repository (e.g. GitHub). See the Nature Portfolio guidelines for submitting code & software for further information.

Data

Policy information about availability of data

All manuscripts must include a data availability statement. This statement should provide the following information, where applicable:

- Accession codes, unique identifiers, or web links for publicly available datasets
- A description of any restrictions on data availability
- For clinical datasets or third party data, please ensure that the statement adheres to our policy

All data are available in the paper and source data files.
Expression vectors developed in this study are available from Addgene:
Expression plasmids for human codon-optimized increased-fidelity (i.e. high-fidelity) SpCas9 variants: B-Sniper SpCas9 (#207361), B-HiFi SpCas9 (#207362) HypaR-SpCas9 (Addgene #126757), B-HypaR-SpCas9 (Addgene #126764, B-evoSpCas9-V495M (#207363), B-evoSpCas9- N515Y (#207364), B-evoSpCas9- E526K (#207365), B-evoSpCas9- Q661R (#207366), B-HeFSpCas9-A661R (#207367), B-HeFSpCas9- A695Q (#207368), B-HeFSpCas9-A848K (#207369) , B-HeFSpCas9-A926Q (#207370) , B-HeFSpCas9-A1003K (#207371) , B-HeFSpCas9-A1060R (#207372)
Expression of increased-fidelity (i.e. high-fidelity) SpCas9 variants in bacterial cells: WT SpCas9 (#207373), Sniper SpCas9 (#207374), Blackjack SpCas9 (#207375), HiFi SpCas9 (#207376), B-Sniper SpCas9 (#207377), B-HiFi SpCas9 (#207378), eSpCas9 (#207379), eSpCas9-plus (#207380), SpCas9-HF1-plus (#207381), SpCas9-HF1 (#207382), B-eSpCas9 (#207383), HypaSpCas9 (#207384), B-SpCas9-HF1 (#207385), B-HypaSpCas9 (#207386), HypaR-SpCas9 (#207387), B-HypaR-SpCas9 (#207388), evoSpCas9 (#207389), B-evoSpCas9 (#207390), HeFSpCas9 (#207391), B-HeFSpCas9 (#207392).
The deep sequencing data are available in NCBI Sequence Read Archive (accession number: SUB13704883).

Human research participants

Policy information about studies involving human research participants and Sex and Gender in Research.

Reporting on sex and gender	N/A
Population characteristics	N/A
Recruitment	N/A
Ethics oversight	N/A

Note that full information on the approval of the study protocol must also be provided in the manuscript.

Field-specific reporting

Please select the one below that is the best fit for your research. If you are not sure, read the appropriate sections before making your selection.

[x] Life sciences [ ] Behavioural & social sciences [ ] Ecological, evolutionary & environmental sciences

For a reference copy of the document with all sections, see nature.com/documents/nr-reporting-summary-flat.pdf

Life sciences study design

All studies must disclose on these points even when the disclosure is negative.

Sample size	No statistical methods were used to predetermine or justify sample size, but each condition was performed in tripliate which is generally accepted sample size for similar gene editing experiments.
Data exclusions	No data were excluded.
Replication	Independent replicates (n=3) were performed. All attempts at replication were successful.
Randomization	No randomization was necessary for the experimental design.
Blinding	No blinding was necessary, as no subjective assessments were required.

Reporting for specific materials, systems and methods

We require information from authors about some types of materials, experimental systems and methods used in many studies. Here, indicate whether each material, system or method listed is relevant to your study. If you are not sure if a list item applies to your research, read the appropriate section before selecting a response.

Materials & experimental systems

n/a	Involved in the study
☒	☐ Antibodies
☐	☒ Eukaryotic cell lines
☒	☐ Palaeontology and archaeology
☒	☐ Animals and other organisms
☒	☐ Clinical data
☒	☐ Dual use research of concern

Methods

n/a	Involved in the study
☒	☐ ChIP-seq
☐	☒ Flow cytometry
☒	☐ MRI-based neuroimaging

Eukaryotic cell lines

Policy information about cell lines and Sex and Gender in Research

Cell line source(s)	Cells employed in the studies are the following: HEK293 (Gibco 293-H cells), GM08207 (Coriell Cell Repositories, Simian virus 40-transformed XP-D fibroblast) were obtained from their suppliers.<br>N2a.dd-EGFP (a neuro-2a mouse neuroblastoma cell line developed by us containing a single integrated copy of an EGFP-DHFR[DD] [EGFP-folA dihydrofolate reductase destabilization domain] fusion protein coding cassette originating from a donor plasmid with 1,000 bp long homology arms to the Prnp gene driven by the Prnp promoter (Prnp.HA-EGFP-DHFR[DD]), N2a.EGFP and HEK-293.EGFP (both cell lines containing a single integrated copy of an EGFP cassette driven by the Prnp promoter) cells were generated by us from the following cell lines: HEK293 (Gibco 293-H cells), N2a (neuro-2a mouse neuroblastoma cells, ATCC, CCL-131).
Authentication	Cell lines were authenticated by their respective suppliers or cloned from those cell lines.
Mycoplasma contamination	Cell lines were regularly tested negative for mycoplasma.
Commonly misidentified lines (See ICLAC register)	No commonly misidentified cell lines were used.

Flow Cytometry

Plots

Confirm that:

☒ The axis labels state the marker and fluorochrome used (e.g. CD4-FITC).

☒ The axis scales are clearly visible. Include numbers along axes only for bottom left plot of group (a 'group' is an analysis of identical markers).

☒ All plots are contour plots with outliers or pseudocolor plots.

☒ A numerical value for number of cells or percentage (with statistics) is provided.

Methodology

Sample preparation	Cells were washed with PBS and after that trypsinized for 1 min. After cells were separated (confirmed by microscope), completed DMEM was added.
Instrument	Flow cytometry analysis was carried out using an Attune NxT Acoustic Focusing Cytometer (Applied Biosystems by Life Technologies).
Software	Attune Cytometric Software v.2.7.0 was used for data analysis.
Cell population abundance	Single cells were gated based on side and forward light-scatter parameters and a total of 5,000 to 10,000 viable single cell events were acquired in all experiments.
Gating strategy	Negative controls (cells not transfected) were used to establish GFP and mCherry +/- gates. GFP and mCherry signals were detected using the 488 (for GFP) and 561 nm (for mCherry) diode laser for excitation, and the 530/30 (GFP) and 620/15 (mCherry) filter for emission.

☒ Tick this box to confirm that a figure exemplifying the gating strategy is provided in the Supplementary Information.
