# Supplementary material for: A cleavage rule for selection of increased-fidelity SpCas9 variants with high efficiency and no detectable off-targets
Source: Nat Commun. 2023 Sep 16;14:5746. doi: 10.1038/s41467-023-41393-5 (PMC10505190; doi:10.1038/s41467-023-41393-5)
Supplement: Supplementary file 3 — Description of Additional Supplementary Files [file 41467_2023_41393_MOESM3_ESM.pdf]

Title: Supplementary Data 1

Description: This file contains target, primer sequences and mutations of Cas9 variants.

Title: Supplementary Data 2

Description: This file contains EGFP disruption data.

Title: Supplementary Data 3

Description: This file contains EGFP disruption processed data.

Title: Supplementary Data 4

Description: This file contains heatmap, G-mean and ROC curve data.

Title: Supplementary Data 5

Description: This file contains processed NGS data.

Title: Supplementary Data 6

Description: This file contains GUIDE-seq processed data.

Title: Supplementary Data 7

Description: This file contains Kim et al. processed dataset.

Title: Supplementary Data 8

Description: This file contains *in vitro* experiment data.

Title: Supplementary Data 9

Description: This file contains statistical details.
